# How to Handle Hard-to-Purify Polymers: Ammonium Sulfate Precipitation of rPEG as a Prototype for Amorphous and Flexible Polymers

**DOI:** 10.1021/acsmacrolett.6c00187

**Published:** 2026-06-02

**Authors:** Lea Simon, Philip Dreier, Holger Frey

**Affiliations:** Department of Chemistry, 9182 Johannes Gutenberg University Mainz, 55128 Mainz, Germany

## Abstract

This work introduces a simple and effective method for purifying both amorphous and semicrystalline, flexible hydrophilic polymers by using ammonium sulfate precipitation (ASP), a technique commonly used in protein purification. ASP exploits the salting-out effect at high ionic strength to precipitate water-soluble polymers, leaving other substances in solution. We successfully purified amorphous and semicrystalline copolymers of ethylene oxide (EO) and racemic glycidyl methyl ether (GME), known as randomized PEG (rPEG), using ASP. The workup was performed from organic reaction media, yielding isolation yields of up to 97% while efficiently removing the solvent, low molar mass impurities and salts resulting from the synthesis. High purity of the isolated polymers was confirmed by NMR spectroscopy, size-exclusion chromatography, mass spectrometry and atomic absorption spectrometry. Owing to its cost-effectiveness and rapid implementation, ASP represents a viable alternative to conventional purification methods such as dialysis and high-pressure liquid chromatography and has the potential to purify a wide range of hydrophilic polymers beyond rPEG, e.g., for biomedical applications.

## Graphical abstract

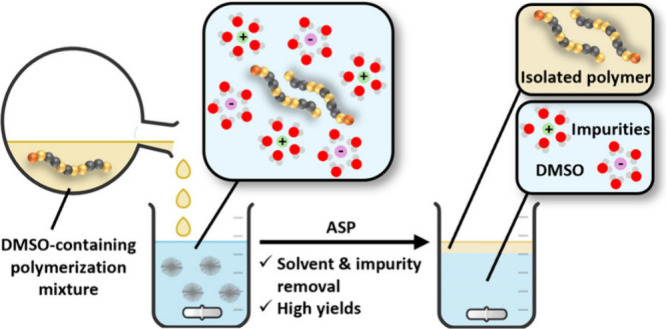

Developing effective polymer purification methods, especially for novel polymeric materials, is crucial in both academia and industry. Ideally, purification methods should be scalable, cost-effective and compatible with a wide range of polymers. Precipitation in a nonsolvent is a common technique for purifying polymers from low-molar-mass impurities. However, selecting an appropriate solvent–nonsolvent system can be challenging, especially for amorphous and flexible, i.e., low *T*_g_ polymers, when precipitation is not driven by crystallization or aided by a high glass transition temperature.^1,2^ Other purification techniques, such as preparative high-pressure liquid chromatography (HPLC) or dialysis, are effective for purifying polymers.^3–7^ However, they are often time-intensive and expensive, as specialized equipment is required. Dialysis can also lead to significant material loss depending on the molar mass and molar mass distribution of the crude polymer. Moreover, the use of high-boiling polar solvents, such as dimethyl sulfoxide (DMSO), *N*-methyl-2-pyrrolidone (NMP), *N,N*-dimethylformamide (DMF), and *N,N*-dimethylacetamide (DMAc), is often required for the synthesis of different biomedically relevant polymers, albeit making their efficient removal challenging yet essential for high purity applications. In this Letter, we introduce ammonium sulfate precipitation (ASP), a method commonly used to purify proteins from aqueous solutions, as an alternative to conventional workup methods for amorphous and semicrystalline polymers.^8^ This method takes advantage of the strong salting-out effect of ammonium sulfate, along with its affordability and easy handling. Water-soluble polymers such as poly­(ethylene glycol) (PEG) and poly­(*N*-isopropylacrylamide) (PNIPAM) demonstrate salting-out behavior in aqueous solutions similar to that of proteins, resulting in salt-induced phase separation and precipitation.^9–11^ Because of this similar response, ASP has been described for the fractionation of water-soluble polymers in aqueous systems, although only a few studies have reported its use to date.^12,13^ However, ASP has not yet been applied to the workup of polymers directly from organic reaction media. Motivated by this, we exemplarily evaluated whether randomized poly­(ethylene glycol) (rPEG) samples, i.e., ideally random, statistical copolymers of ethylene oxide (EO) and glycidyl methyl ether (GME) (P­(EO-*co*-GME)), of various molar masses and compositions can be directly purified from a DMSO-containing polymerization mixture using ASP. rPEG structures, constitutional isomers of PEG,^14^ serve as an ideal model system as the EO/GME ratio dictates the polymer’s structural and thermal properties (Figure 1). rPEGs with low GME content exhibit semicrystalline behavior. Increasing the GME fraction above 25 mol % leads to completely amorphous polymers. Thus, it can be investigated whether ASP workup is an effective approach for both amorphous and semicrystalline hydrophilic polymers. Additionally, rPEG purification is of considerable interest as rPEG represents a nonantigenic alternative to PEG for biomedical applications and is also suitable as a PEG substitute for advanced materials that require reduced crystallinity.^13–15^

**Figure 1:**
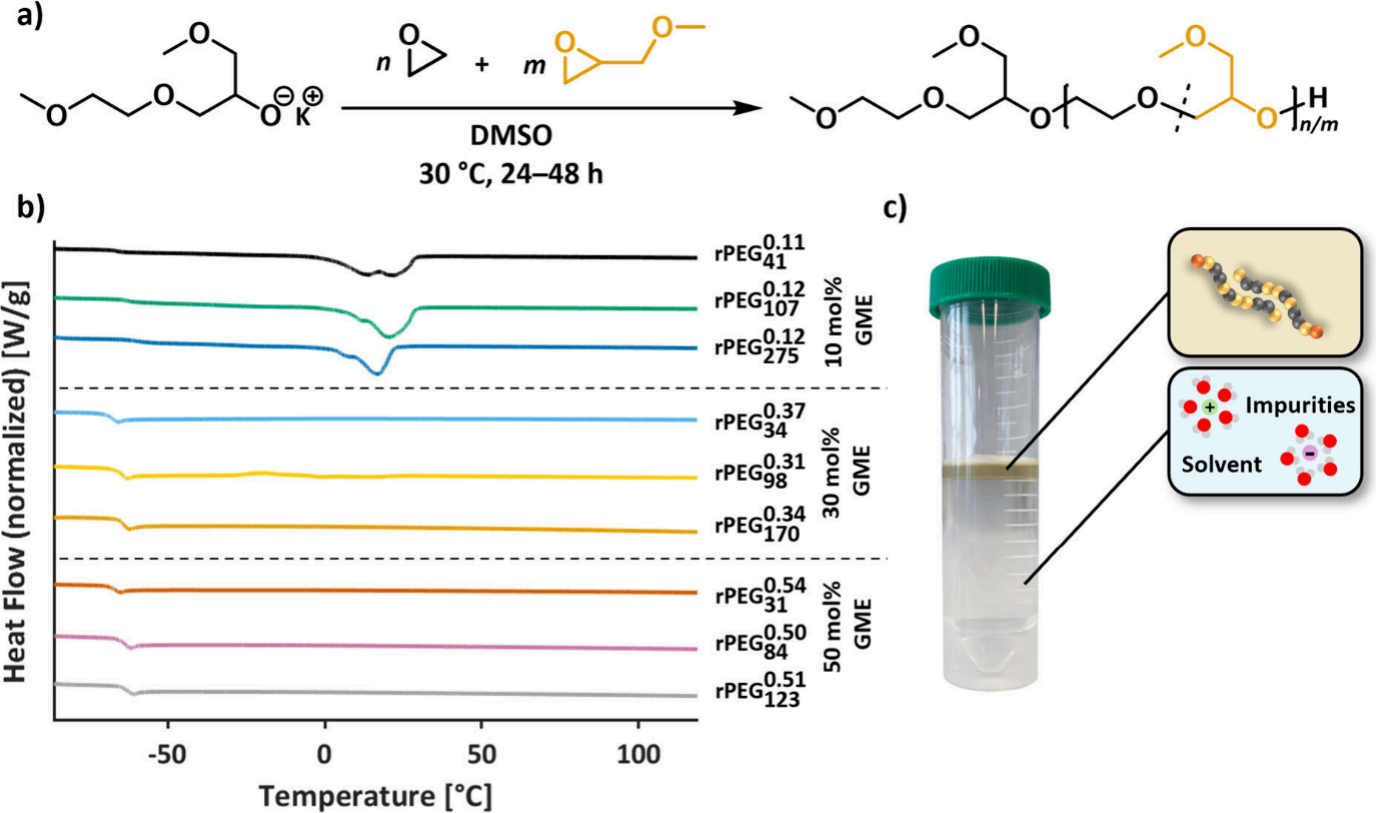
Thermal characterization of systematically varied rPEG_DP_^GME^: (a) Synthesis strategy for rPEG by copolymerization of ethylene oxide and glycidyl methyl ether in DMSO. The termination step is omitted for clarity reasons; (b) Stacked DSC curves of synthesized rPEGs after workup (second heating cycle, exo up); (c) Schematic representation of an aqueous two-phase system formed by salting-out of rPEG using ammonium sulfate (DP = degree of polymerization; GME = mole fraction of GME).

Furthermore, by studying the workup of rPEG, a typical water-soluble material, it is possible to explore whether the ASP workup procedure could potentially be applied to other water-soluble polymers. Examples include poly­(2-oxazoline)­s (POx), hyperbranched or linear polyglycerols (PG), poly­(vinylpyrrolidone) (PVP), polyacrylamides (PAAm), or polysaccharides such as dextran and hyaluronic acid (HA).^16,17^

rPEGs were synthesized by anionic ring-opening polymerization (AROP) of EO and GME, utilizing DMSO as the solvent (Figure 1a). SEC traces of the synthesized rPEGs (Figures S14–S16) exhibit a monomodal and narrow molar mass distribution. The polymer range was chosen to cover a broad scope of rPEGs, with targeted GME content varying from 10 to 50 mol % and molar masses spanning 2 to 10 kg mol^–1^ (Table S1). This enables the investigation of ASP workup of polymers with varying degrees of crystallinity and molar masses, including rPEGs relevant for both biomedical applications and functional materials.^13–15,18^ To evaluate ASP as a workup method, rPEGs were purified directly from the polymerization mixture after termination with acetic acid to remove residual DMSO and low-molar-mass byproducts, including oligomers and residual salts. The method was adapted from established protocols for protein purification and polyether fractionation.^8,12^ The crude sample was diluted with a 25 wt % aqueous ammonium sulfate solution, a concentration selected to induce rPEG precipitation, while remaining below the threshold that would cause ammonium sulfate precipitation in the presence of DMSO. The resulting aqueous two-phase system consists of a polymer-rich upper phase and a salt-rich lower phase (Figure 1c), allowing efficient removal of DMSO, residual salts and small-molecule impurities, as the aqueous lower phase can be readily separated from the polymer-rich upper phase. Purity was further improved through two additional precipitation cycles using a saturated ammonium sulfate solution (43 wt %). Remaining ammonium sulfate was removed by extracting the polymer-rich phase with dichloromethane (DCM), followed by solvent evaporation and lyophilization, yielding pure rPEGs with isolated yields between 65% and 97% (Table 1). The obtained yields were highest for samples with higher molar masses, as phase separation becomes more favorable with increasing degree of polymerization due to the lower Gibbs free energy of mixing.^10,11,19^ Additionally, increased GME content was associated with slightly higher yields, which may be attributed to enhanced inter- and intramolecular hydrophobic interactions imparted by the side chains.^14,20^ Therefore, precipitation efficiency appears to be primarily affected by the molar mass and hydrophilicity of the polymer rather than their crystallinity.

**Table 1:** Comparison of Analytical Results for All rPEG Samples before and after Purification by Ammonium Sulfate Precipitation

sample	*M*_n,crude_^MS^ ^a^ (kg mol^–1^)	*M*_p,crude_^MS^ ^a^ (kg mol^–1^)	*M*_n,final_^MS^ ^a^ (kg mol^–1^)	*M*_p,final_^MS^ ^a^ (kg mol^–1^)	*M*_n,crude_^SEC^ ^b^ (kg mol^–1^)	*M*_n,final_^SEC^ ^b^ (kg mol^–1^)	*Đ*_crude_^SEC^ ^b^	*Đ*_final_^SEC^ ^b^	yield^c^ (%)
rPEG_41_^0.11^	2.0	2.0	2.1	2.1	1.6	1.6	1.08	1.07	65^e^
rPEG_107_^0.12^	5.3	5.3	5.3	5.4	4.1	4.6	1.13	1.06	90
rPEG_275_^0.12^	^d^	^d^	13.6	13.6	12.0	12.0	1.16	1.16	85
rPEG_34_^0.37^	2.1	2.0	2.1	2.1	1.6	1.6	1.07	1.07	75^e^
rPEG_98_^0.31^	5.7	6.0	5.7	5.8	4.1	4.6	1.15	1.05	95
rPEG_170_^0.34^	10.0	10.0	10.1	10.0	8.4	8.5	1.08	1.07	95
rPEG_31_^0.54^	2.0	2.0	2.1	2.0	1.5	1.5	1.08	1.06	84
rPEG_84_^0.50^	5.5	5.6	5.6	5.6	4.2	4.2	1.06	1.06	93
rPEG_123_^0.51^	8.0	8.1	8.2	8.2	5.5	6.0	1.14	1.08	97

a Determined by MALDI-ToF MS (matrix: DCTB; cationizing agent: KTFA).

b Determined by SEC (eluent: DMF; calibration: PEG; RI detector).

c Isolated yield of the polymer, based on the aliquot taken for purification.

d Analysis by MALDI-ToF MS was not feasible.

e Yield loss likely due to low molar mass of polymer.

rPEGs purified by ASP workup were analyzed using NMR spectroscopy, MALDI-ToF mass spectrometry and SEC. Figure 2 shows an exemplary ^1^H NMR spectrum of rPEG_123_^0.51^ at different stages of the purification process. Before purification, the NMR spectrum of this typical crude polymer sample shows signals corresponding to the solvent DMSO, as well as small-molecule impurities in addition to the copolymer signals. The NMR spectrum of the purified sample exclusively displays copolymer signals, indicating high purity. Additionally, ^13^C NMR (Figure S11) and 2D [^1^H,^13^C] NMR spectra (Figures S12 and S13) further confirm the absence of low molar mass impurities. ^1^H NMR spectra from the other rPEG samples (Figures S2–S10) confirm the purity of all samples after ASP workup. Sample compositions before and after purification were characterized using MALDI-ToF MS and SEC (Table 1). The comparative analysis of the data indicates no significant changes in molar mass distribution for all samples, i.e., no undesired fractionation is observed. Comparison of the MALDI-ToF mass spectra of the crude and purified rPEG samples (Figures S18–S26) confirms that the detected species remain consistent throughout purification, in line with the absence of oligomers in the crude samples. Additional mass peaks observed in the MALDI-ToF mass spectra of some crude samples can be attributed to counterions that are absent in the purified polymers. Comparison of the SEC elugrams of the purified rPEG samples with the crude samples (Figures S14–S16) shows no peak broadening or signs of degradation, confirming that the ASP workup does not affect the sample composition for the investigated samples. The apparent decrease in dispersity observed in some purified samples compared to the crude material likely results from the removal of lower-molar-mass polymer fractions formed by delayed initiation or by trace amounts of water in the monomer acting as initiators. This is exemplarily shown by comparing SEC elugrams of one rPEG sample (rPEG_98_^0.31^) at different purification stages (Figure S17). The comparison suggests that lower-molar-mass polymers tend to remain in the aqueous phase during ASP, likely due to their higher aqueous solubility.^10,19^

**Figure 2:**
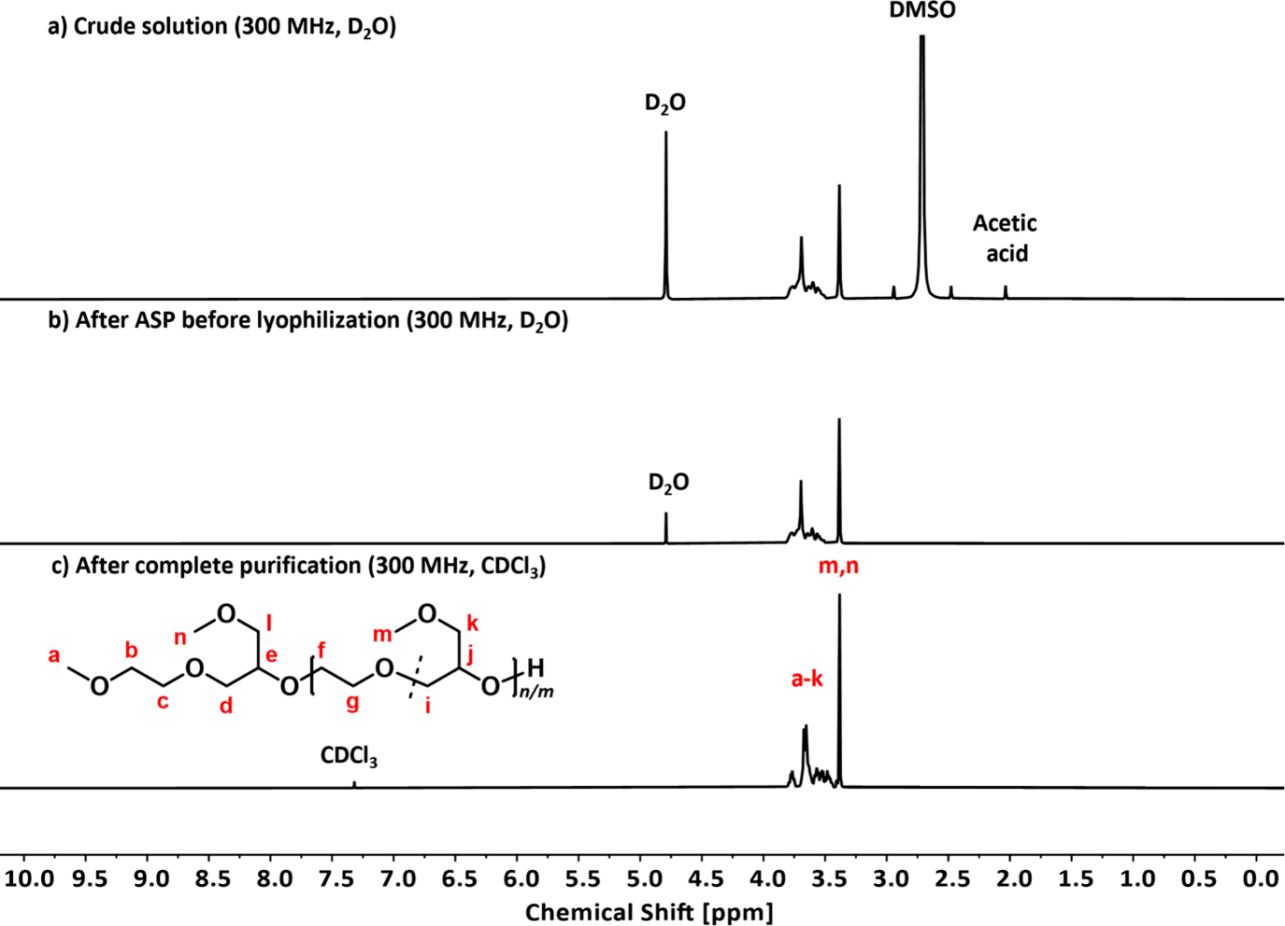
Typical ^1^H NMR spectra of rPEG_123_^0.51^ at different stages of the purification process: (a) the crude polymerization mixture (300 MHz, D_2_O); (b) after ammonium sulfate precipitation workup but before lyophilization (300 MHz, D_2_O); (c) after complete purification (300 MHz, anhydrous CDCl_3_). The change of solvent to anhydrous CDCl_3_ in the final spectrum was intended to demonstrate effective drying of the polymer by lyophilization.

Characterization of the potassium levels of one exemplary purified sample using atomic absorption spectroscopy (AAS) (Figure 3a) indicates that ASP workup considerably reduces potassium levels compared to samples purified by Celite filtration or dialysis. This reduction likely explains the reduced coloration of the ASP-purified aliquot compared to the aliquots purified by dialysis and flash chromatography using Celite (Figure 3b), as ionic residues, such as potassium ions, can cause coloring of the polyethers, probably through interactions with the polyether chain ends.^21^ Overall, the findings demonstrate that ASP effectively removes solvent, small-molecule impurities and ionic residues without altering the composition of the rPEG samples. Additionally, workup with ASP may result in less material loss compared to dialysis or chromatographic purification methods. The ammonium content of the purified samples was quantified by an indophenol-blue (Berthelot) assay adapted from Baethgen and Alley.^22^ ASP-purified polymers show no increase in ammonium levels relative to crude, dialyzed, or Celite-filtered samples (Figure S1), demonstrating that the purification procedure has no adverse effect on residual ammonium content. Lastly, the feasibility of ASP workup of rPEG from other water-miscible organic solvents was investigated. To this end, a representative polymer (rPEG_123_^0.51^) was dissolved in various solvents and subjected to ASP workup. The results (Figure S27 and S28) demonstrate that direct purification from organic polymer solutions is achievable when using solvents such as DMSO, DMF, ethanol and methanol. For other solvents, including those that are not water-miscible, ASP can still be utilized for impurity removal; however, the solvent must be partially reduced or completely removed prior to the purification.

**Figure 3:**
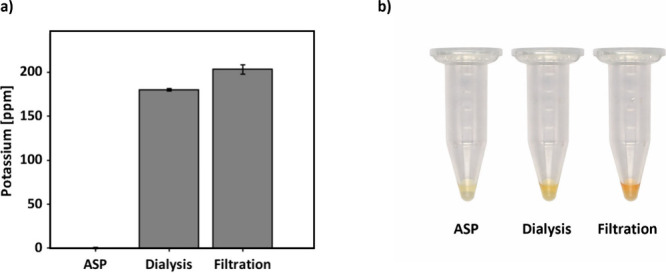
(a) Potassium content and (b) visual appearance of aliquots of sample rPEG_123_^0.51^ after purification by ammonium sulfate precipitation (left), dialysis against water (middle) and removal of DMSO by distillation followed by Celite filtration (right).

In conclusion, we have shown that ASP workup offers a straightforward, rapid and cost-effective method for purifying rPEGs from organic solutions across a wide range of molar masses, including amorphous and semicrystalline materials. Our results demonstrate that ASP workup effectively removes (high boiling) solvents, small-molecule impurities and ionic residues while maintaining the polymers’ molar mass distribution and structural integrity, resulting in high yields after isolation. While this study focused on rPEG as the model system and DMSO as a polar solvent, the results strongly suggest that the ASP workup is broadly applicable to other hydrophilic polymers, such as POx, PG, PVP, PAAm and polysaccharides, as well as to other water-soluble organic solvents. This will be investigated in further studies. In summary, the findings of this study demonstrate that ASP workup is a highly versatile and potent technique with significant potential for polymer purification in both academic research and industrial applications, for instance for high purity biomedical materials.

## Experimental Section

A detailed description of all materials and instruments, including a description of analysis procedures, is referred to in the Supporting Information. Caveat: *Ethylene oxide is a highly flammable and toxic gas; it must be handled by trained researchers and staff!*

### Preparation of Polymers

Randomized PEG (rPEG) samples were synthesized following a previously reported anionic copolymerization procedure.^14^ The GME monomer and the initiator were synthesized as previously described. Detailed experimental conditions are provided in the Supporting Information. Polymer purification was performed by ammonium sulfate precipitation, flash chromatography using Celite or dialysis.

### Purification of Polymers via Ammonium Sulfate Precipitation (ASP)

For polymer purification via ammonium sulfate precipitation, only an aliquot of the polymerization mixture (5 mL) was processed. A 5 mL aliquot of polymerization mixture was combined with two to three-fold the volume of a 25 wt % aqueous ammonium sulfate solution. The resulting turbid mixture was centrifuged (3 min, 4500 rpm) to accelerate phase separation. The lower, salt-rich and polymer-poor phase was removed. The remaining upper phase was diluted with deionized water to the original volume (5 mL). These steps were repeated twice. Due to the reduced solvent (DMSO) content after the first cycle, a saturated ammonium sulfate solution (43 wt %) was used for the second and third precipitation steps. After the final phase separation, the polymer-rich upper phase was diluted with deionized water to the original volume (5 mL) and extracted with dichloromethane (DCM) (3 × 10 mL). The organic phase was dried over magnesium sulfate and the solvent was removed by distillation or under reduced pressure. The purified polymer was further dried by lyophilization. For investigation of the ASP workup from other organic solvents, 250 mg of a previously purified polymer was dissolved in 2.5 mL of the respective solvent and purified according to the procedure described above.

### Purification of Polymers via Flash Chromatography

For polymer purification via flash chromatography, only an aliquot of the polymerization mixture (5 mL) was processed. Workup was adapted from a previously reported procedure.^14^ DMSO was removed under reduced pressure and the residue was redissolved in diethyl ether (15 mL) and toluene (5 mL) under stirring. After 15 min, the mixture was filtered through a dense layer of Celite and the solvents were removed under reduced pressure.

### Purification of Polymers via Dialysis

For polymer purification via dialysis, only an aliquot of the polymerization mixture (5 mL) was processed. DMSO was removed under reduced pressure. The resulting residue was dissolved in deionized water and dialyzed against deionized water for 3 days using a dialysis membrane (*Spectrum Laboratories*) with a molar mass cutoff of 1 kDa. The purified polymer was dried by lyophilization.

## Supplementary Material



## Notes

Additional analytical data (NMR, SEC, MALDI-ToF MS), experimental procedures and methods and supplementary text and tables (PDF)
