# Supplementary material for: How to Handle Hard-to-Purify Polymers: Ammonium Sulfate Precipitation of rPEG as a Prototype for Amorphous and Flexible Polymers
Source: ACS Macro Lett. 2026 Jun 2;15(6):859–63. doi: 10.1021/acsmacrolett.6c00187 (PMC13276894; doi:10.1021/acsmacrolett.6c00187)
Supplement: Supplementary file 1 [file mz6c00187_si_001.pdf]

Supporting Information for

How to Handle Hard-to-Purify Polymers: Ammonium Sulfate Precipitation of rPEG as a Prototype for Amorphous and Flexible Polymers

Authors: Lea Simon[1], Philip Dreier[1], and Holger Frey[1]*

[1]Department of Chemistry, Johannes Gutenberg University Mainz, 55128 Mainz, Germany

*Corresponding author. E-Mail: hfrey@uni-mainz.de

Supporting Information

Supplementary Text

rPEG Sample Description:[14]

Throughout this manuscript and the Supporting Information, the randomized PEG (rPEG) sample composition is described with:

$$\text{rPEG}_{DP}^{f}$$

where f is the molar fraction of glycidyl methyl ether (GME) in the sample, and DP is the total degree of polymerization.

Calculation of incorporated GME content and total degree of polymerization:[14]

The GME content and DP in the rPEG samples were not determined by end-group analysis of the respective ^{1}H NMR spectra due to overlap between methoxy groups from the initiator and methoxy methylene side groups. Instead, the GME content was quantified using a combination of MALDI-ToF mass spectrometry and ^{1}H NMR spectroscopy. Therefore, the number-average molar mass ($M_{\text{n,MALDI}}$) and the ratio of the integrals of backbone signals (I_{backbone}, from 4.10 to 3.45 ppm) to methoxy group signals (I_{MeO}, from 3.45 to 3.30 ppm) were obtained via MALDI-ToF MS and ^{1}H NMR, respectively. The values derived from these measurements were used to calculate DP_{GME}, the molar GME content ($mol\%_{\text{GME}}$), and the total DP for the rPEG samples by selecting the parameter set that best fit the experimental data.

$$\frac{I_{\text{backbone}}}{I_{\text{MeO}}} = \frac{5 \cdot DP_{\text{GME}} + 4 \cdot DP_{\text{EO}}}{3 \cdot DP_{\text{GME}}} \qquad \textbf{(S1)}$$

$$DP_{\text{EO}} = \frac{DP \cdot (100\text{-}mol\%_{\text{GME}})}{100} \qquad \textbf{(S2)}$$

$$DP_{\text{GME}} = \frac{DP \cdot mol\%_{\text{GME}}}{100} \qquad \textbf{(S3)}$$

$$DP = \frac{M_{\text{n,MALDI}}\text{-}M_{\text{Initiator}}}{\left(M_{\text{EO}} \cdot \left(\frac{(100\text{-}mol\%_{GME})}{100}\right)\right) + \left(M_{\text{GME}} \cdot \left(\frac{mol\%_{GME}}{100}\right)\right)} \qquad \textbf{(S4)}$$

Synthesis of randomized PEG (rPEG):

Caveat: Ethylene oxide is a highly flammable and toxic gas; it must be handled by trained researchers and staff! rPEG was synthesized according to the standard procedure described in a previous publication.[14] The copolymerization is described for the example of $\text{rPEG}_{41}^{0.11}$. This standardized protocol was used across all rPEG samples, with the EO/GME ratio adjusted to achieve the desired molar amount of GME and the specified molar mass (Table S1).

Potassium *tert*-butoxide (KO^tBu, 0.10 g, 0.90 mmol, 0.98 eq.) was dissolved in stabilizer-free THF (7.5 mL) containing five drops of Milli-Q® water in a flame-dried 100 mL anionic polymerization flask equipped with a septum and magnetic stir bar under a counter-flow of argon. A solution of 1-methoxy-3-(2-methoxyethoxy)propan-2-ol (MMEPOH, 0.15 g, 0.92 mmol, 1.00 eq.) in benzene (7.5 mL) was added dropwise while stirring and under argon flow. The mixture was stirred for 5 min, after which the apparatus was carefully evacuated while stirring. Residual water was removed azeotropically overnight under high vacuum at 55 °C. The resulting dried initiator salt was dissolved under static vacuum in anhydrous DMSO (10.0 mL). The solution was frozen using an ethanol–liquid nitrogen bath (-78 °C) before adding dry glycidyl methyl ether (GME, 0.25 mL, 2.76 mmol, 3.00 eq.) via syringe through the septum. Ethylene oxide (EO, 1.50 mL, 33.13 mmol, 36.00 eq.) was condensed into a graduated ampule at -78 °C and transferred to the frozen reaction mixture via cryotransfer under static vacuum. The mixture was allowed to warm to room temperature and stirred for 1 day at 30 °C under static high vacuum. Polymerization was terminated by gentle exposure to air. Acetic acid (0.21 mL) was added, and the mixture was stirred for 30 min. Polymer purification was performed by ammonium sulfate precipitation (ASP). The polymer was obtained as a colorless, slightly yellow liquid.

^1H NMR (300 MHz, $CDCl_3$ δ): 3.85–3.45 (m, polyether backbone), 3.36 (s, OCH_3) ppm.

Materials and Instruments

Reagents

All chemicals and solvents were purchased from *Acros Organics*, *Fisher Scientific*, *Roth*, *TCI Chemicals*, *Sigma-Aldrich*, *Avantor VWR*, and *abcr GmbH* unless otherwise noted. Ethylene oxide (EO) was obtained from *Air Liquide*. Deuterated solvents were purchased from *Deutero GmbH*. For the anionic ring-opening polymerization (AROP), tetrahydrofuran (THF) was passed through basic aluminum oxide, and glycidyl methyl ether (GME) was dried over CaH_2 before cryo-transfer for polymerization.

Instrumentation

Nuclear Magnetic Resonance (NMR) Spectroscopy:

^{1}H NMR spectra were recorded on a Bruker Avance III HD 300 spectrometer at a frequency of 300 MHz (^{1}H) or a Bruker Avance III HD 400 spectrometer with 400 MHz (^{1}H) or 101 MHz (^{13}C), with internal referencing to residual proton signal of the deuterated solvent. All spectra were acquired at room temperature. Spectra were processed and analyzed using *MestReNova 14.3.3* from *Mestrelab Research.*

Size Exclusion Chromatography (SEC):

SEC analysis was performed on an *Agilent 1100 series* HPLC system equipped using a HEMA 300/100/40 column set. Detection was performed using a refractive index (RI) detector (Agilent G1362A) and a UV254 detector (Agilent G1314A). The eluent was DMF containing 1 mg mL^{-1} anhydrous LiBr at a flow rate of 1.0 mL min^{-1}. The column oven and RI detector were maintained at 50 °C. Calibration was performed with PEG standards (*PSS Polymer Standards Service GmbH*). Samples (2 mg mL^{-1} in DMF/LiBr with one drop of toluene) were filtered through 0.45 µm PTFE filters, and 100 µL were injected. Toluene was used as an internal standard. Data were analyzed using *PSS WinGPC Unichrom V8.31*.

Matrix-assisted Laser Desorption Ionization Time-of-Flight (MALDI-ToF) Mass Spectrometry:

MALDI-ToF MS measurements used a *Bruker autoflex maX MALDI-ToF/ToF* with a smartbeam-II laser at 337 nm. Spectra were acquired with the software *Bruker flexControl 3.4* and analyzed using *Bruker flexAnalysis 3.4* and *Bruker polytools 1.31*. The ionization salt was KTFA, and the matrix was DCTB. Polymers were dissolved in chloroform (10 mg mL^{-1}), and 20 µL of the solution was mixed with 20 µL of the matrix solution. Then, 5 µL of a 0.1 M salt in methanol was added, and 1 µL of the mixture was spotted on an MTP 384 ground steel target plate. Solvent evaporation was complete before measurement. All measurements were conducted in linear mode.

Differential Scanning Calorimetry (DSC):

DSC measurements were carried out using a *DSC250* instrument from *TA Instruments*. Samples with a mass of approximately 5–10 mg were sealed in *Tzero* aluminum pans with a hole. The samples were first heated to 120 °C and held isothermally for 10 min to erase thermal history, followed by cooling at a rate of 10 °C min^{-1} to −90 °C and an isothermal hold at −90 °C for 10 min. Subsequently, DSC measurements were performed from −90 °C to 120 °C at a heating rate of 10 °C min^{-1}. Data from the second heating cycle were analyzed using *TRIOS* software from *TA Instruments*.

Polymer Analysis

Ammonium Determination:

Ammonium concentrations were quantified by a microplate-based indophenol-blue (Berthelot) assay adapted from Baethgen and Alley.[22] In this assay, ammonium reacts with hypochlorite in alkaline medium to form monochloramine, which subsequently reacts with salicylate in the presence of sodium nitroprusside to yield a green–blue indophenol dye. The absorbance of the dye is proportional to the ammonium concentration and was measured at λ = 650 nm using a *BMG* Labtech *FLUOstar® Omega* microplate reader.

The salicylate reagent was prepared by dissolving sodium salicylate (6.8 g), trisodium citrate (5.0 g), sodium tartrate (5.0 g), and sodium nitroprusside (0.025 g) in Milli-Q® water and diluting to 100 mL. A 60 g L^{-1} NaOH solution was prepared by dissolving 6.0 g NaOH in 100 mL of Milli-Q® water. The hypochlorite reagent was freshly prepared by diluting 1 mL of 10% sodium hypochlorite solution to 50 mL with the NaOH solution. A 10 mM ammonium stock solution was prepared by dissolving ammonium sulfate (0.066 g) in 100 mL of Milli-Q® water. Calibration standards (0–200 mM ammonium) were prepared by serial dilution of the ammonium stock solution with Milli-Q® water. For analysis, 40 µL of diluted sample (0.1 mg Polymer per mL^{-1}) or standard was transferred into a 96-well plate, followed by the addition of 80 µL salicylate reagent and 80 µL hypochlorite reagent. The plate was mixed thoroughly and incubated at room temperature for 45 min before absorbance was measured at 650 nm.

Potassium Determination:

Perkin Elmer 5100 ZL atom absorption/emission (AA/E) spectrometer equipped with a Zeeman furnace module was used to determine the potassium concentrations. The measurements were performed using a Varian hollow cathode lamp at the 766.5 nm potassium absorption line and a lamp current of 12 mA. Calibration standards in the range of 0.5–2.0 mg L^{-1} were prepared by dilution of a stock solution with K concentration of 1 g L^{-1} with Milli-Q® water. Polymer samples were diluted with

Milli-Q® water as required to ensure measurements within the instrument's linear range. For each sample, three consecutive measurements were collected and averaged.

Supporting Tables

Table S1. Summary of polymerization degrees and copolymer compositions of the synthesized rPEGs, determined by ^{1}H NMR spectroscopy, MALDI-ToF MS, and SEC.

Sample	$M_n^{theo(a)}$/ kg mol^{-1}	mol%$_{GME}^{theo(a)}$	$M_n^{MS(b)}$/ kg mol^{-1}	$M_p^{MS(b)}$/ kg mol^{-1}	$DP_{n_{total}}^{MS(c)}$	$mol\%_{GME}^{NMR(c)}$	$M_n^{SEC(d)}$/ kg mol^{-1}	$Đ^{SEC(d)}$
rPEG$_{41}^{0.11}$	2.0	10%	2.1	2.1	41	11%	1.6	1.07
rPEG$_{107}^{0.12}$	6.0	10%	5.3	5.4	107	12%	4.6	1.06
rPEG$_{275}^{0.12}$	10.0	10%	13.6	13.6	275	12%	12.0	1.16
rPEG$_{34}^{0.37}$	1.9	30%	2.1	2.1	34	37%	1.6	1.07
rPEG$_{98}^{0.31}$	6.0	30%	5.7	5.8	98	31%	4.6	1.06
rPEG$_{170}^{0.34}$	9.9	30%	10.1	10.0	170	34%	8.5	1.07
rPEG$_{31}^{0.54}$	2.0	50%	2.1	2.0	31	54%	1.5	1.06
rPEG$_{84}^{0.50}$	6.1	50%	5.6	5.6	84	50%	4.2	1.06
rPEG$_{123}^{0.51}$	10.1	50%	8.2	8.2	123	51%	6.0	1.08

a) Calculated based on the monomer equivalents used.
b) Determined using MALDI-ToF MS (matrix: DCTB, ionization reagent: KTFA).
c) Determined by combining M_n^{MS} and integral ratios from ^{1}H NMR spectroscopy (300 MHz, $CDCl_3$, 25 °C).
d) Determined by SEC (eluent: DMF, calibration: PEG, detector: RI).

Table S2. Thermal properties of synthesized rPEGs (second heating cycle, 10 K min^{-1}).

Sample	T_g / °C	T_m / °C	ΔH_m / J g^{-1}
rPEG$_{41}^{0.11}$	−65	22	49
rPEG$_{107}^{0.12}$	−63	21	53
rPEG$_{275}^{0.12}$	−60	17	37
rPEG$_{34}^{0.37}$	−69	-	-
rPEG$_{98}^{0.31}$	−65	−1	4
rPEG$_{170}^{0.34}$	−62	-	-
rPEG$_{31}^{0.54}$	−68	-	-
rPEG$_{84}^{0.50}$	−65	-	-
rPEG$_{123}^{0.51}$	−64	-	-

Supporting Figures

Ammonium Determination

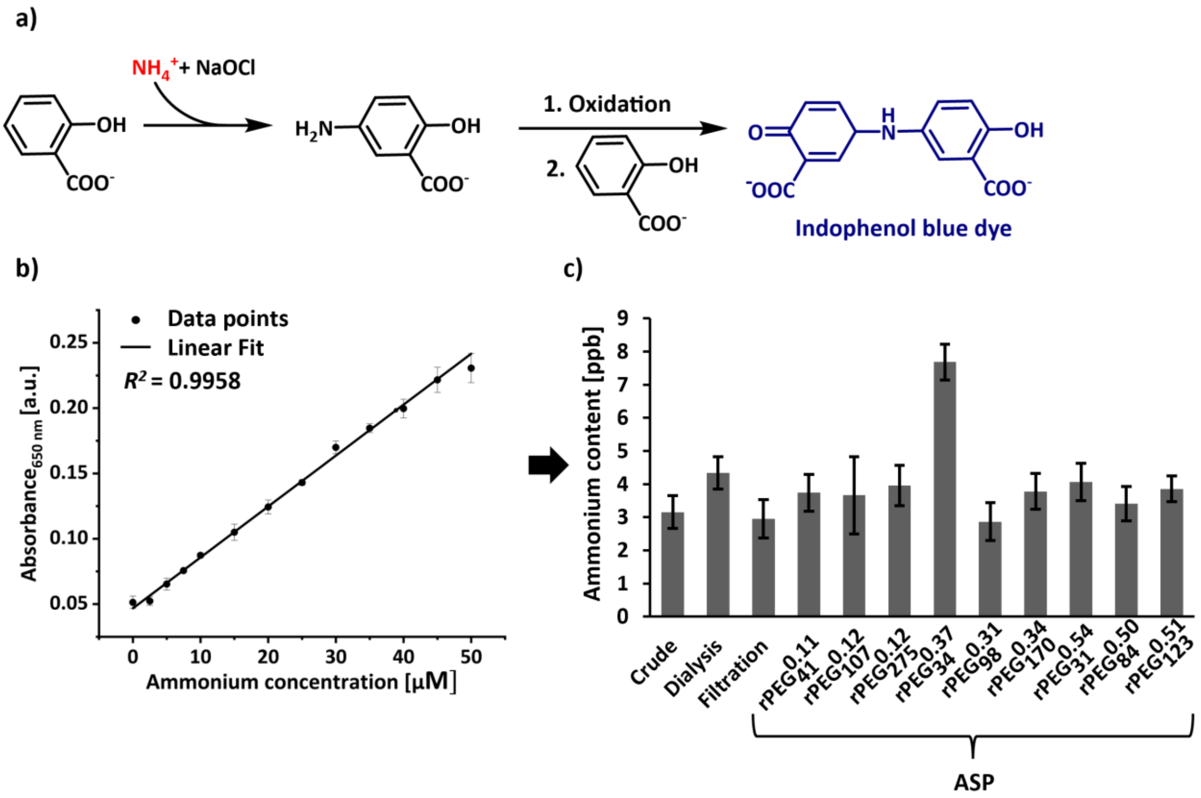

Figure S1. Ammonium concentration in polymer samples purified by ASP workup compared to alternative workup methods. a) Reaction scheme illustrating the conversion of salicylic acid in the presence of ammonium to form the indophenol blue derivate; b) External calibration curve for ammonium concentration, recorded at 650 nm using a microplate reader; c) Ammonium content of polymer aliquots of sample $rPEG_{123}^{0.51}$ in the crude state, after purification by dialysis against water and after DMSO removal followed by Celite® filtration, compared to rPEG samples purified via ASP. The slightly higher ammonium level observed for sample $rPEG_{34}^{0.37}$ is likely an outlier caused by minor handling variations during work-up, such as insufficient washing or drying of the polymer, which may have led to residual ammonium sulfate remaining in the material. This effect was not observed in any of the other rPEG samples.

NMR Spectra

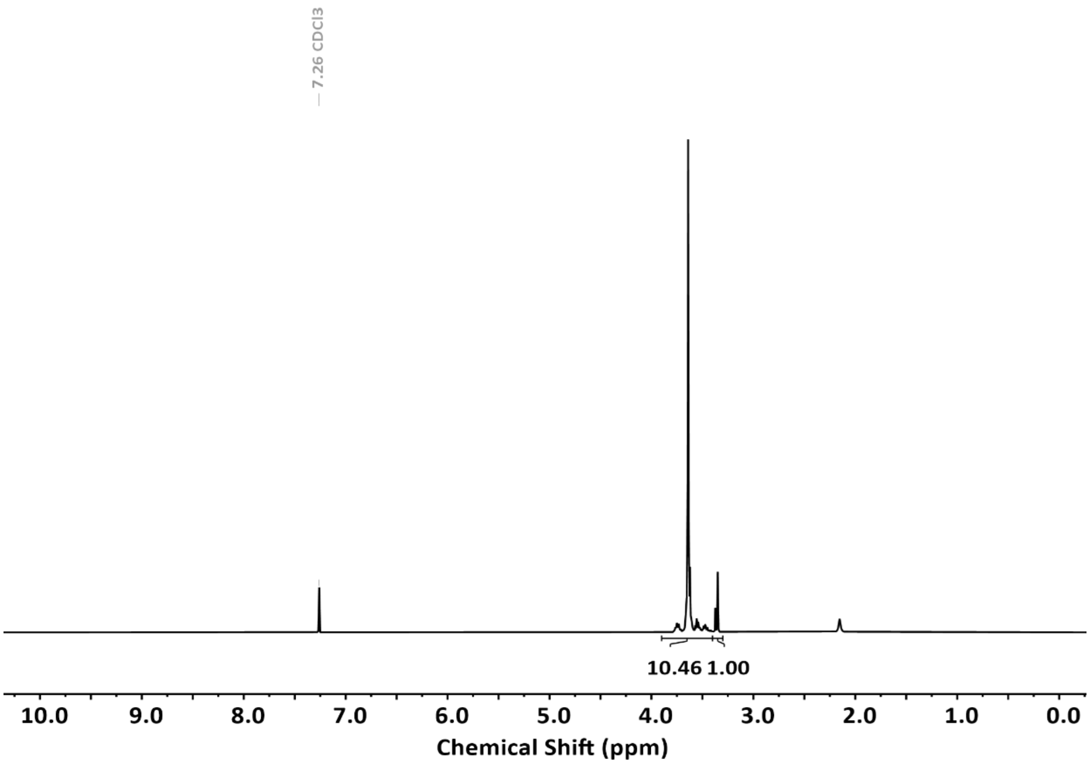

Figure S2. ^{1}H NMR spectrum of rPEG$_{41}^{0.11}$ after purification (300 MHz, $CDCl_3$).

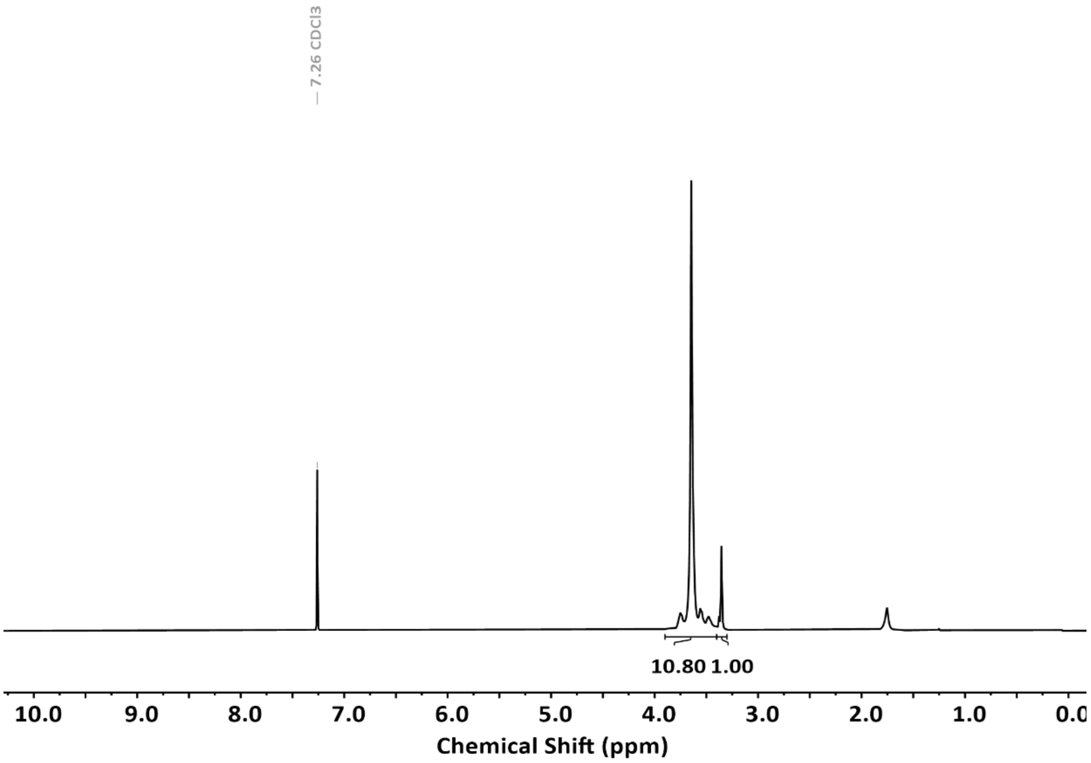

Figure S3. ^{1}H NMR spectrum of rPEG$_{107}^{0.12}$ after purification (300 MHz, $CDCl_3$).

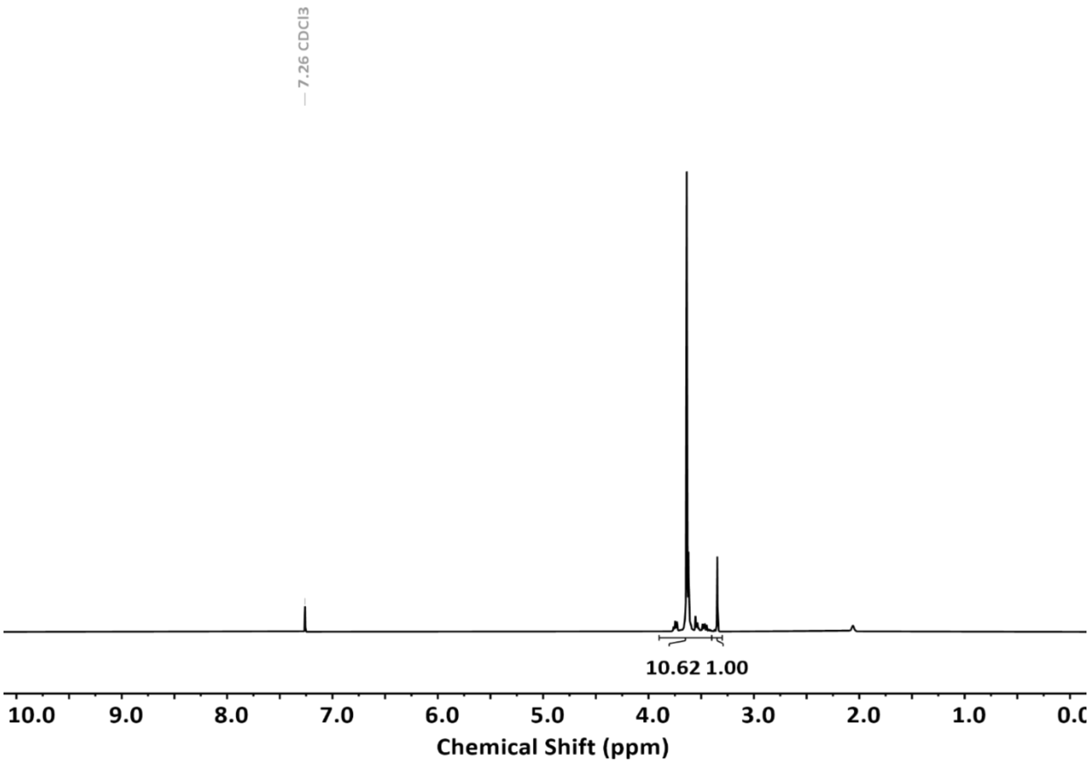

Figure S4. ^{1}H NMR spectrum of rPEG$^{0.12}_{275}$ after purification (300 MHz, $CDCl_3$).

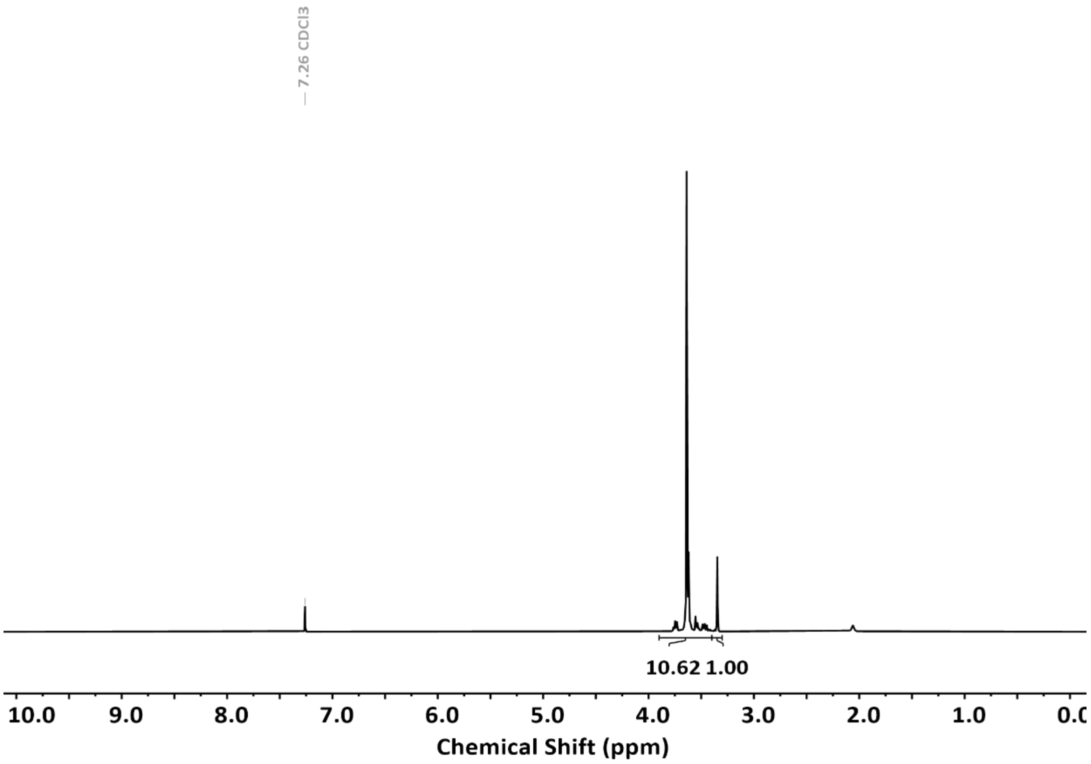

Figure S5. ^{1}H NMR spectrum of rPEG$^{0.37}_{34}$ after purification (300 MHz, $CDCl_3$).

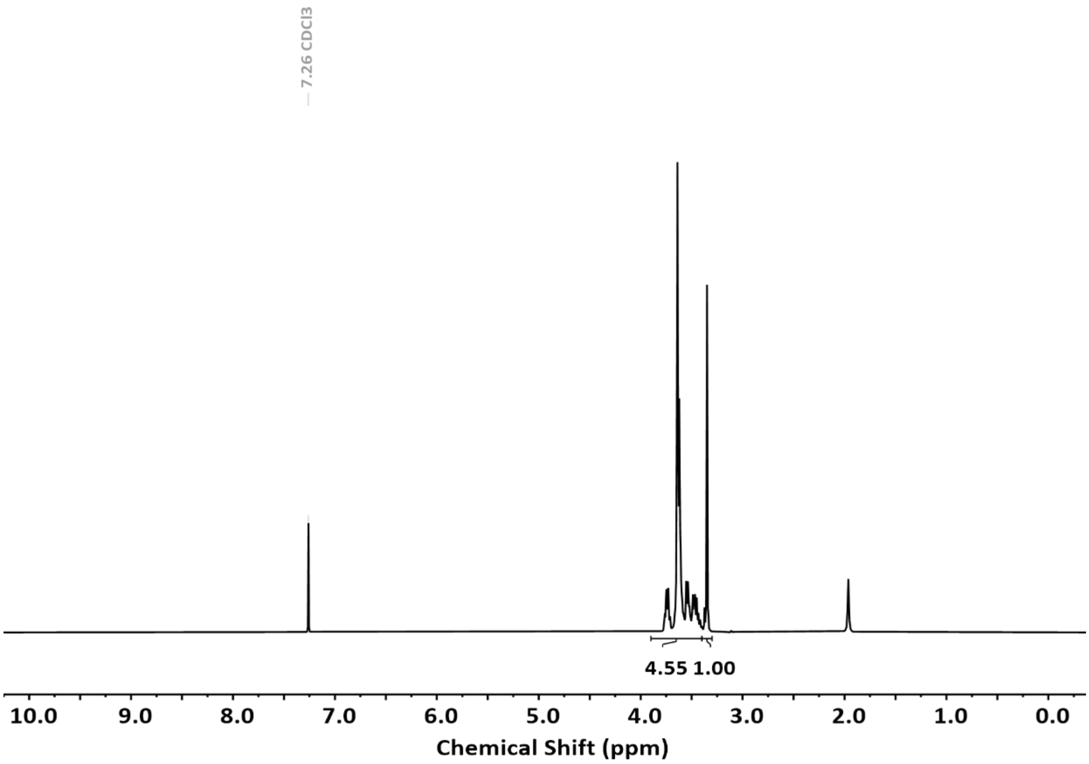

Figure S6. ^{1}H NMR spectrum of $rPEG_{98}^{0.31}$ after purification (300 MHz, $CDCl_3$).

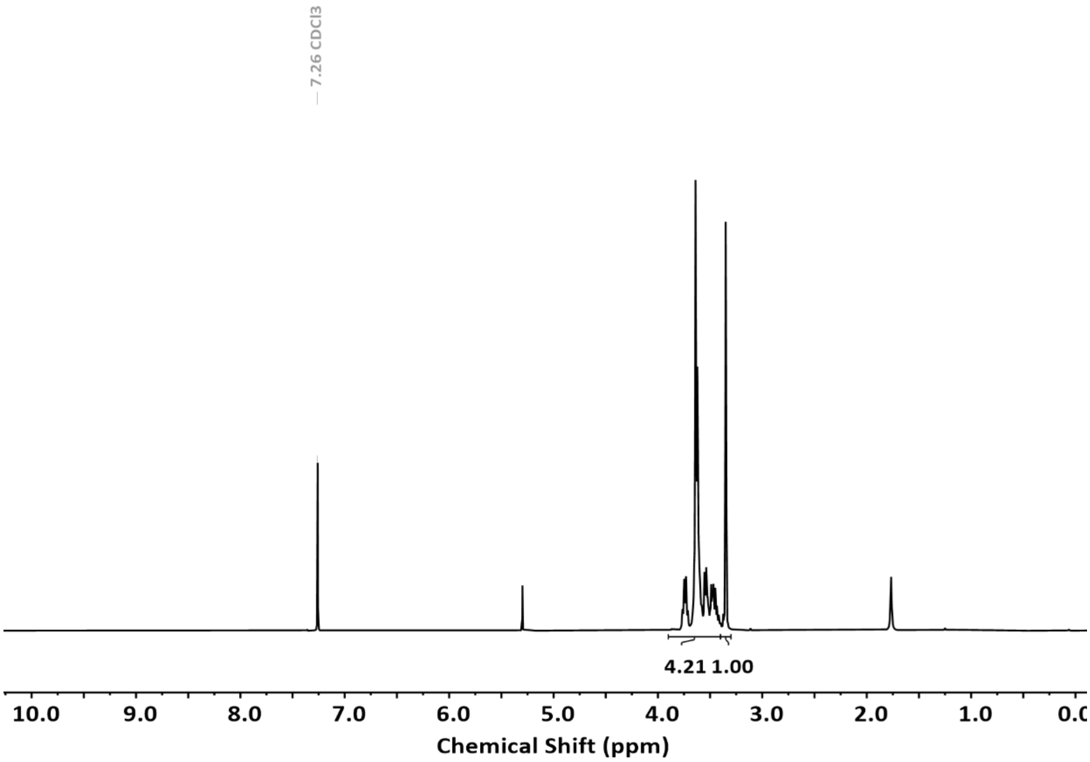

Figure S7. ^{1}H NMR spectrum of $rPEG_{170}^{0.34}$ after purification (300 MHz, $CDCl_3$).

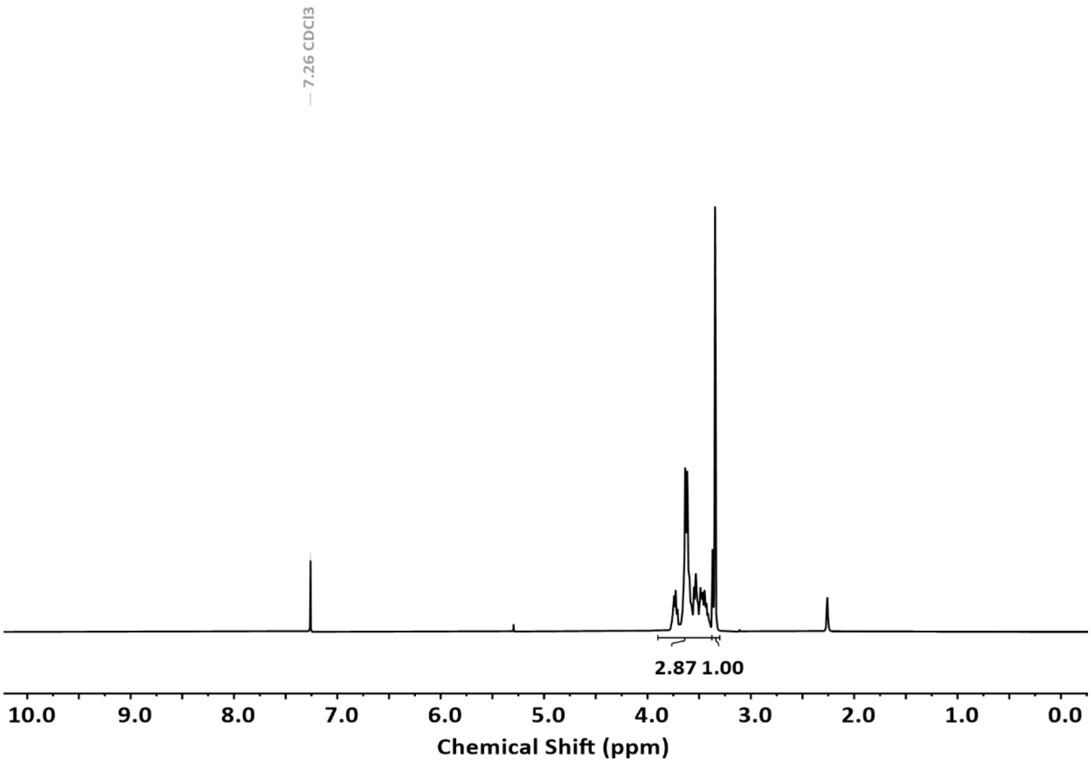

Figure S8. ^{1}H NMR spectrum of $rPEG_{31}^{0.54}$ after purification (300 MHz, $CDCl_3$).

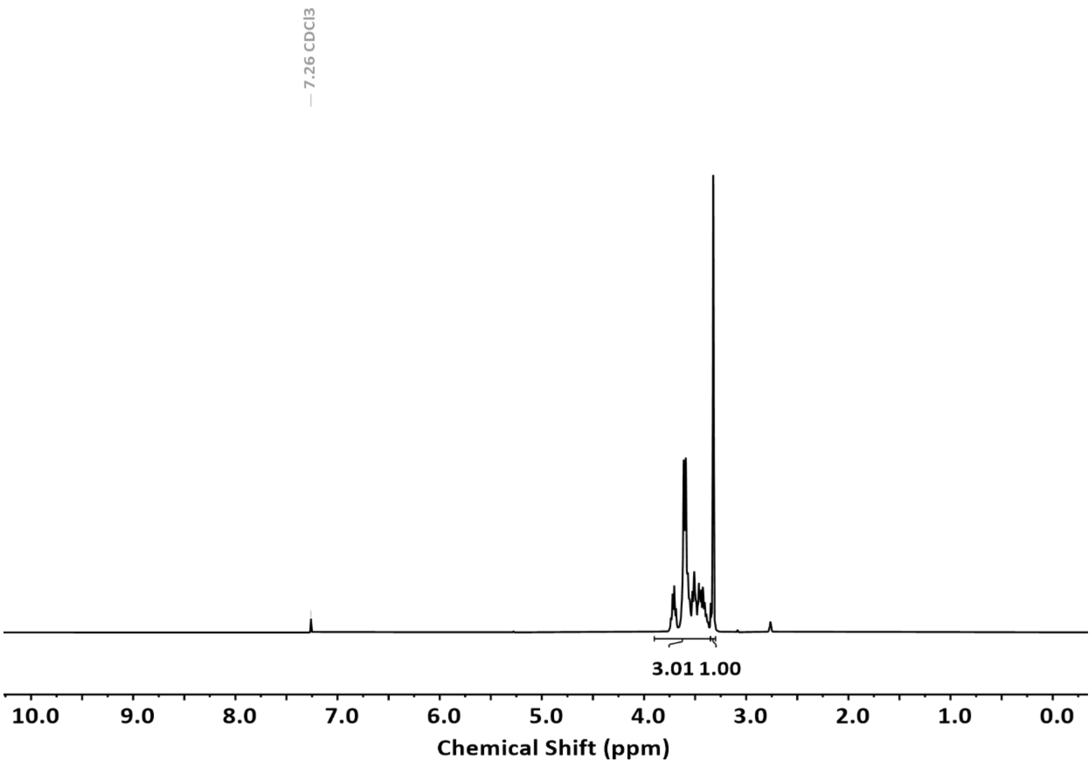

Figure S9. ^{1}H NMR spectrum of $rPEG_{84}^{0.50}$ after purification (300 MHz, $CDCl_3$).

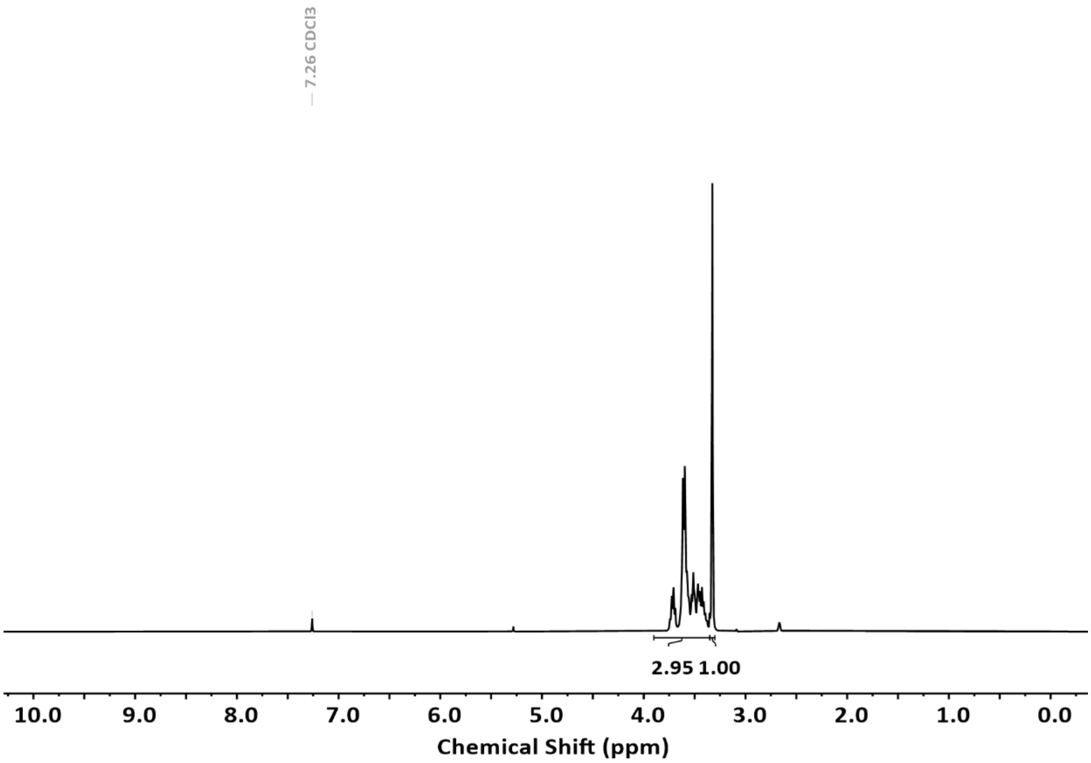

Figure S10. ^{1}H NMR spectrum of $rPEG_{123}^{0.51}$ after purification (300 MHz, $CDCl_3$).

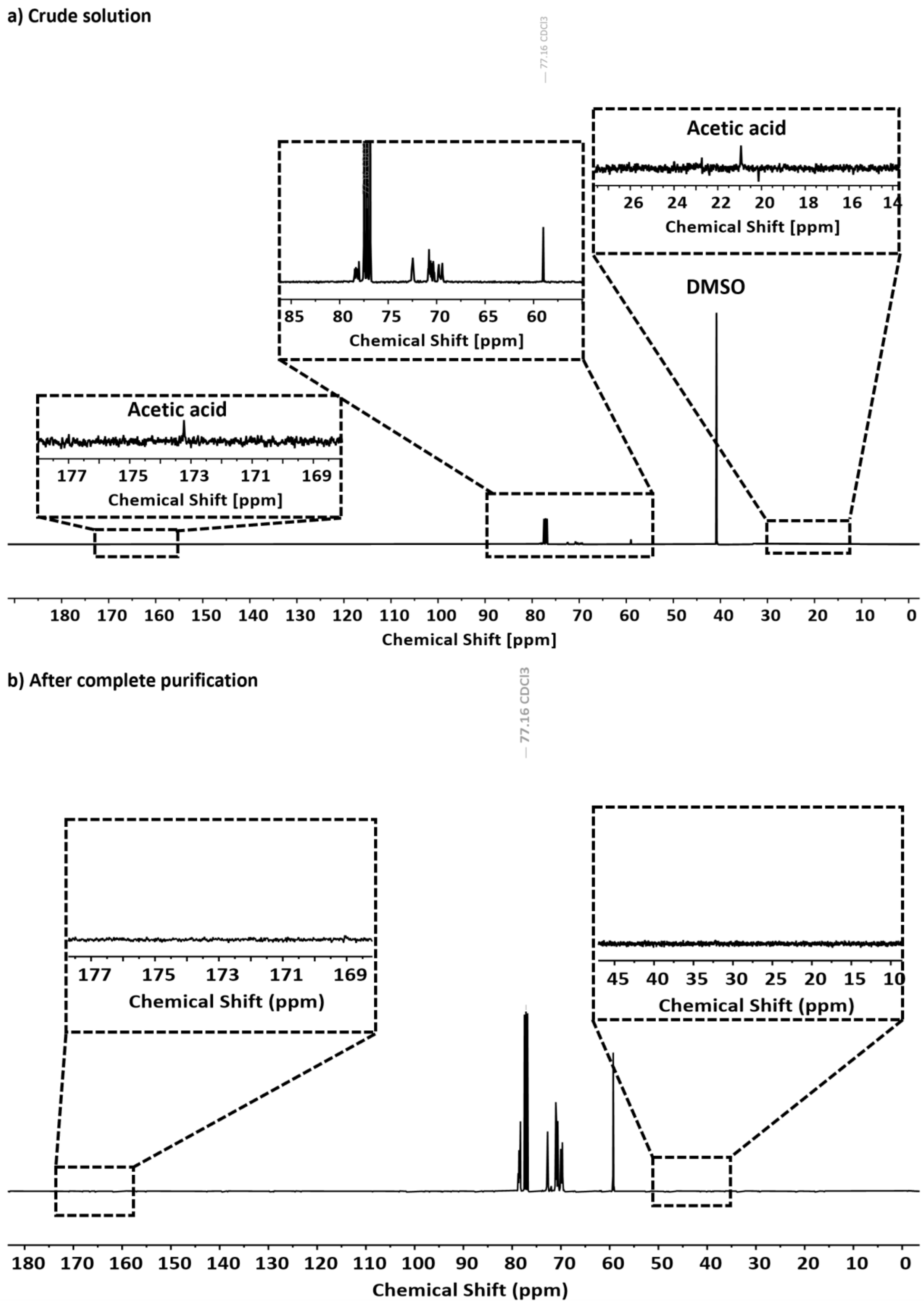

Figure S11 ^{13}C NMR spectrum of $rPEG_{123}^{0.51}$ (101 MHz, $CDCl_3$): (a) crude polymer solution before purification and (b) purified polymer after complete purification. The magnified region in spectrum (b) shows no residual signals attributable to DMSO or acetic acid, confirming the high purity of the polymer.

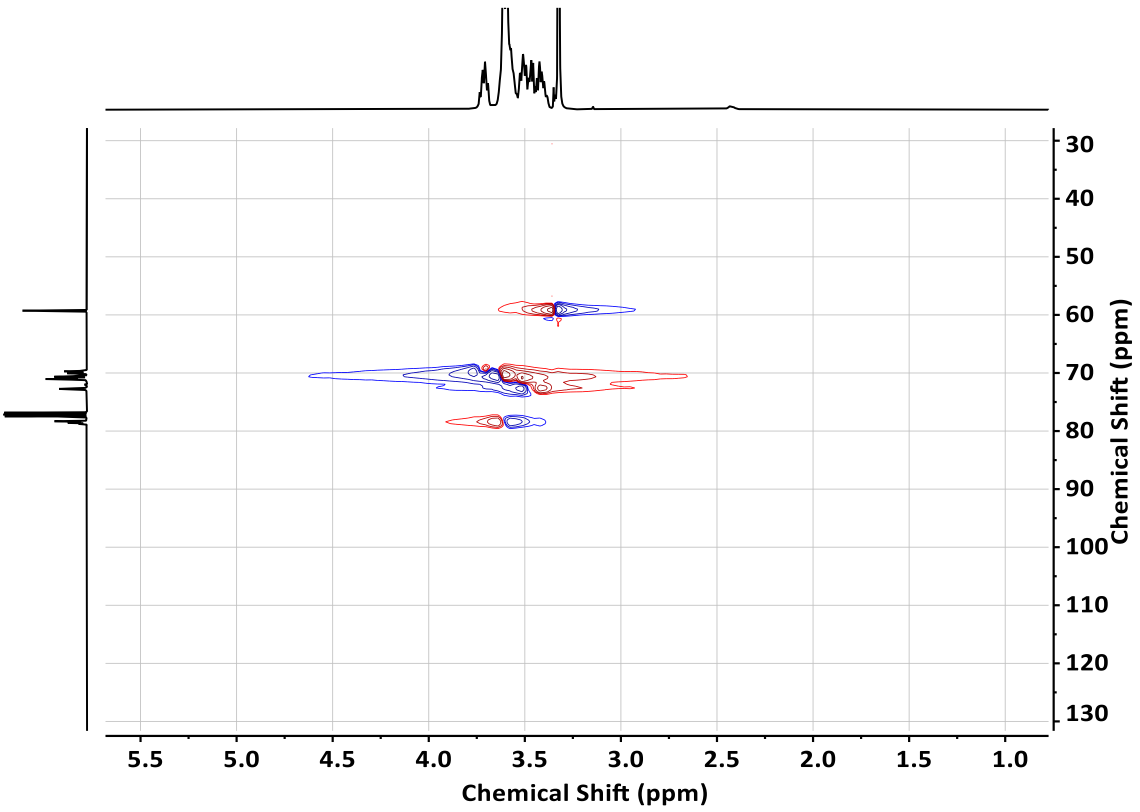

Figure S12. [^{1}H, ^{13}C] HSQC NMR spectrum of rPEG$^{0.51}_{123}$ after purification (400 MHz, $CDCl_3$).

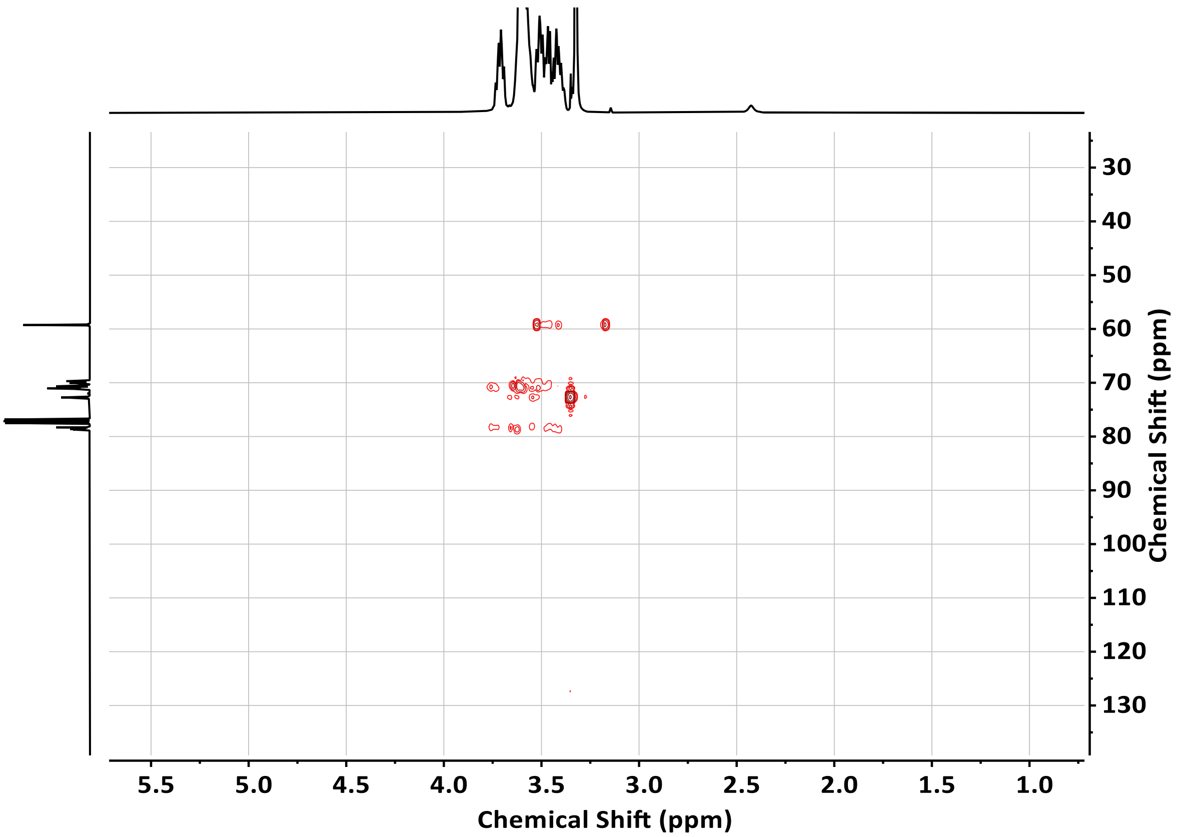

Figure S13. [^{1}H, ^{13}C] HMBC NMR spectrum of rPEG$^{0.51}_{123}$ after purification (400 MHz, $CDCl_3$).

SEC Traces

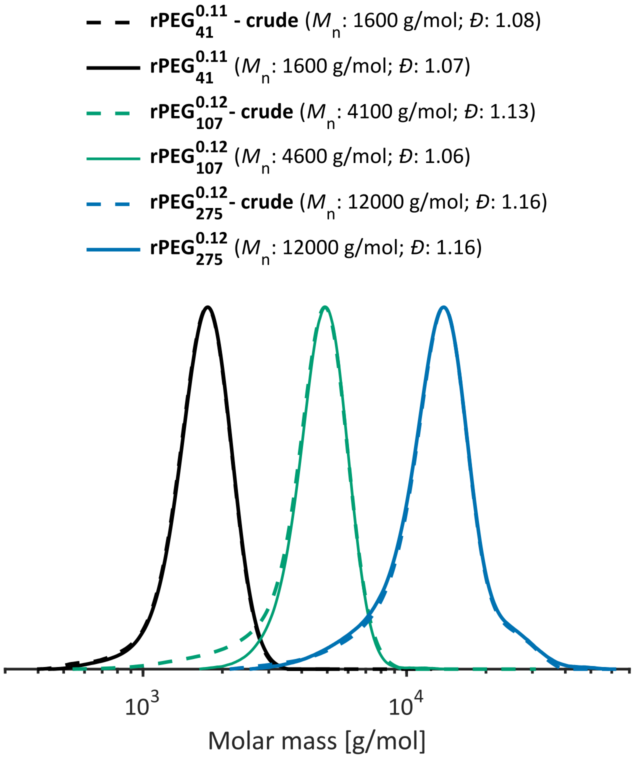

Figure S14. SEC traces of rPEG samples with 10 mol% GME targeted (DMF, calibration: PEG, detector: RI).

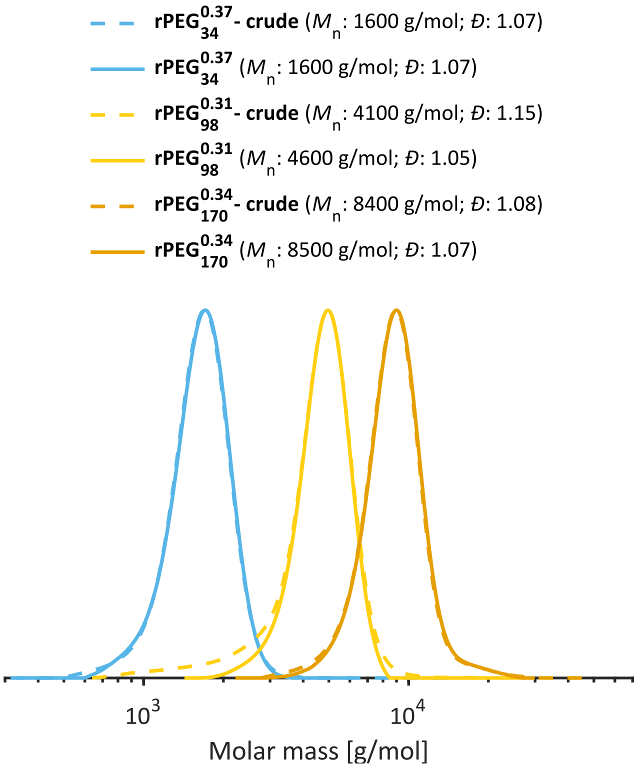

Figure S15. SEC traces of rPEG samples with 30 mol% GME targeted (DMF, calibration: PEG, detector: RI).

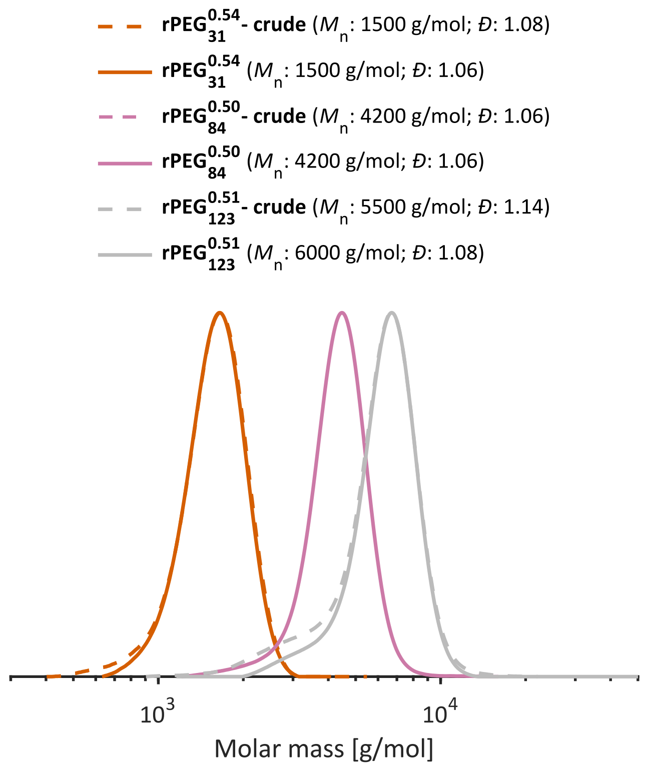

Figure S16. SEC traces of rPEG samples with 50 mol% GME targeted (DMF, calibration: PEG, detector: RI).

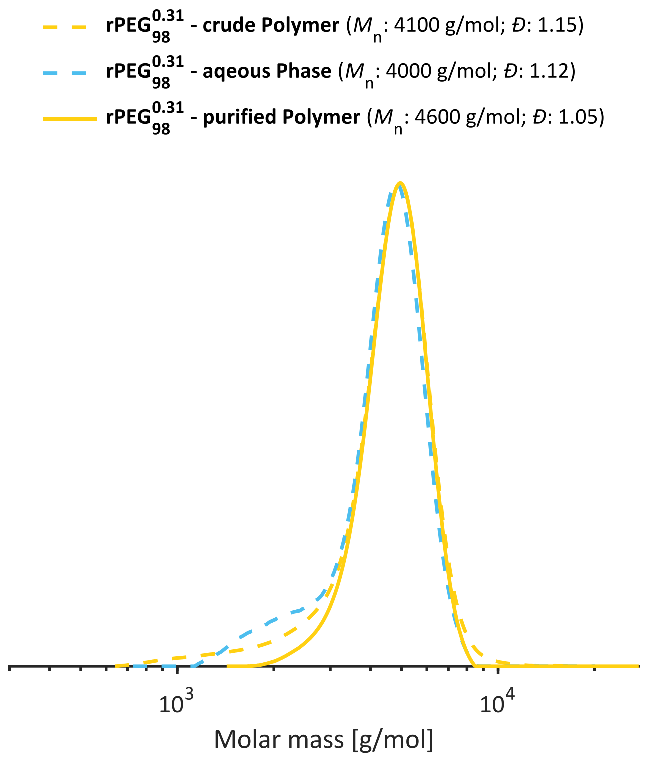

Figure S17. Exemplary comparison of SEC traces of $rPEG_{98}^{0.31}$ sample during purification process: crude polymer (yellow, dotted), residue in the aqueous phase after ammonium sulfate precipitation extracted with DCM (blue, dotted), and purified polymer (yellow, solid). (Eluent: DMF; calibration: PEG standards; detector: RI).

MALDI-ToF Mass Spectra

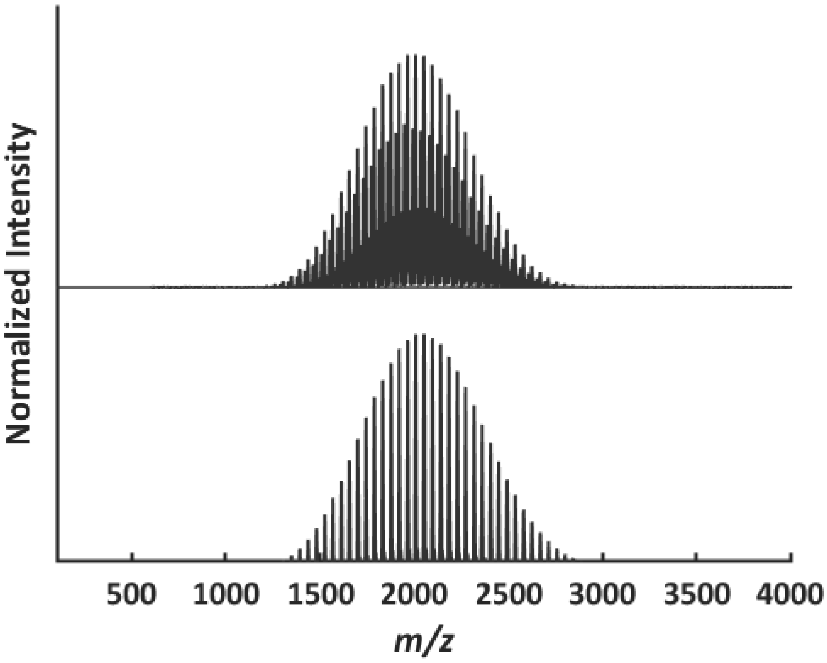

Figure S18. Stacked MALDI-TOF mass spectra of rPEG$_{41}^{0.11}$: (i) crude sample and (ii) sample after purification via ASP. Additional species observed in the crude sample can be attributed to different ionic counterions, without indicating any change in the polymer composition.

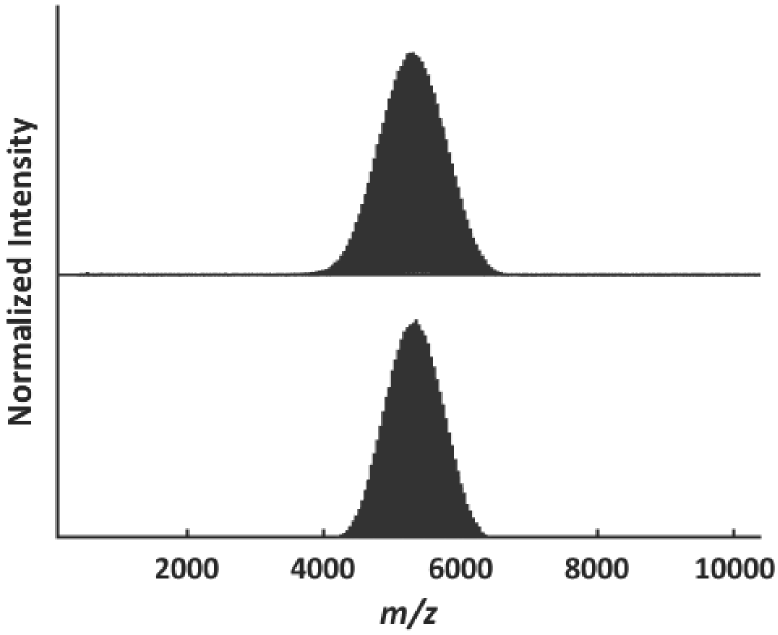

Figure S19. Stacked MALDI-TOF mass spectra of rPEG$_{107}^{0.12}$: (i) crude sample and (ii) sample after purification via ASP.

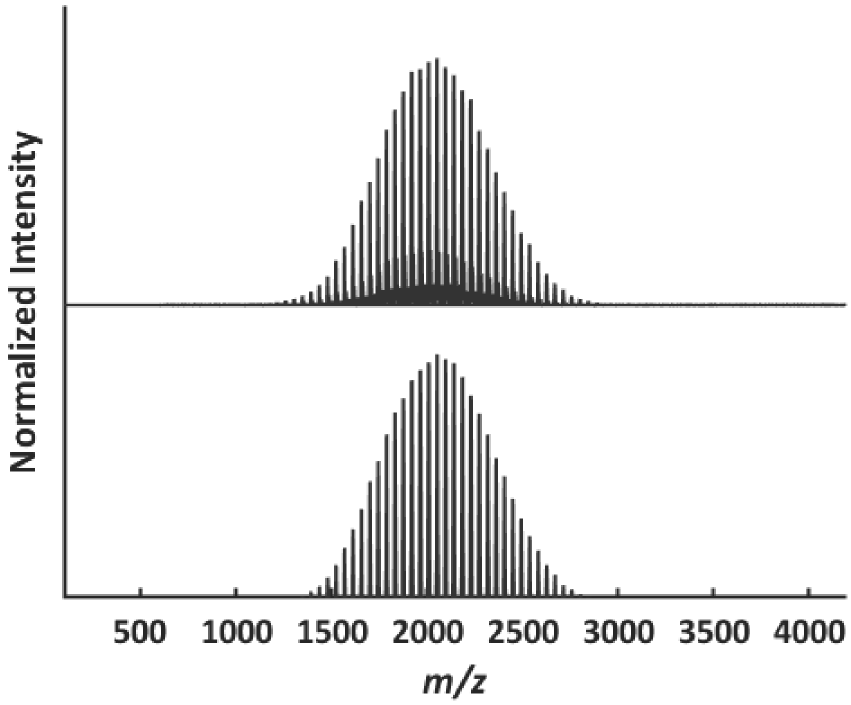

Figure S20. Stacked MALDI-TOF mass spectra of rPEG$_{34}^{0.37}$: (i) crude sample and (ii) sample after purification via ASP. Additional species observed in the crude sample can be attributed to different ionic counterions, without indicating any change in the polymer composition.

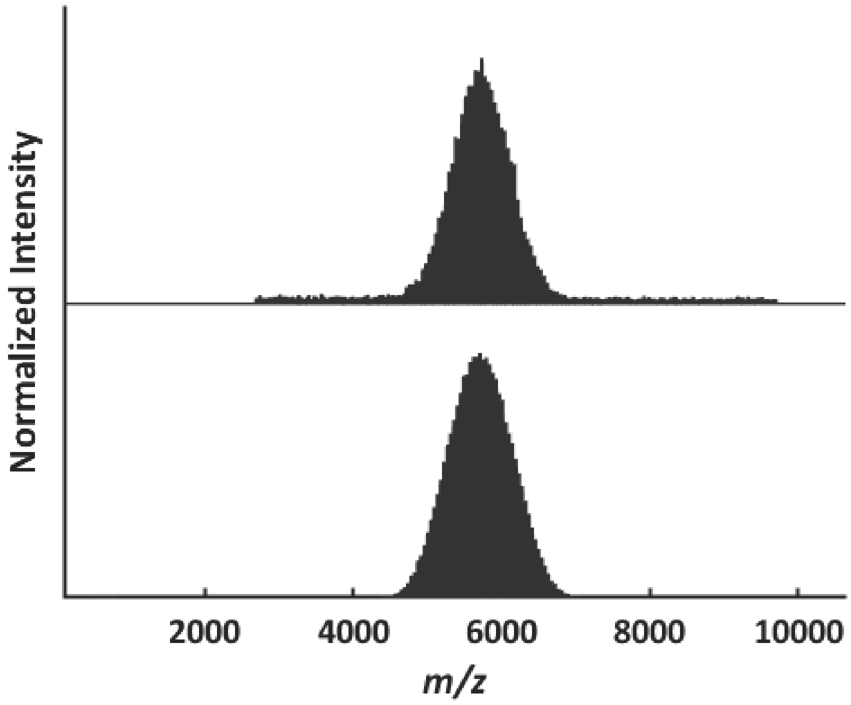

Figure S21. Stacked MALDI-TOF mass spectra of rPEG$_{98}^{0.31}$: (i) crude sample and (ii) sample after purification via ASP.

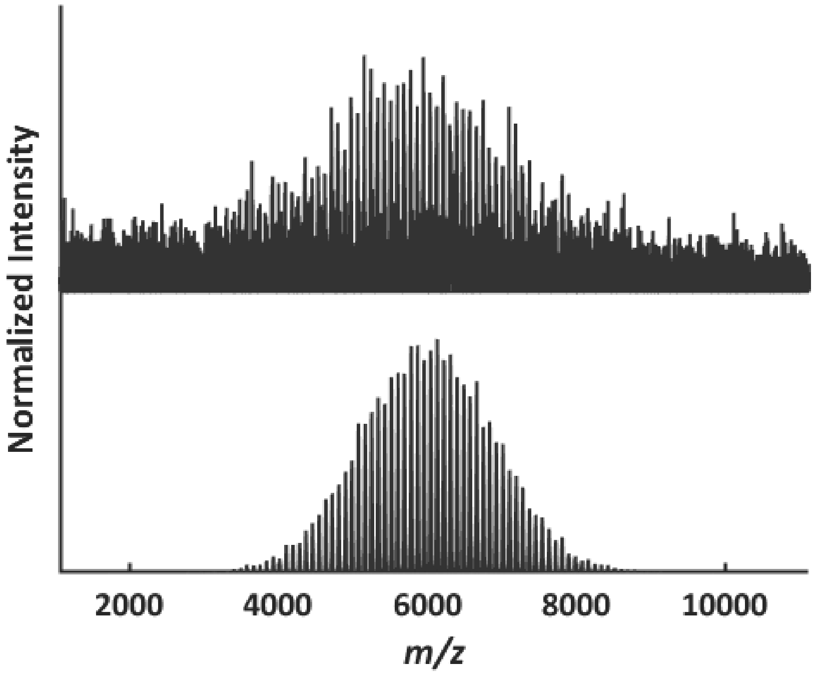

Figure S22. Stacked MALDI-TOF mass spectra of rPEG$^{0.34}_{170}$: (i) crude sample and (ii) sample after purification via ASP. Mass spectrum of the crude polymer sample showing reduced mass resolution at higher m/z values due to residual impurities.

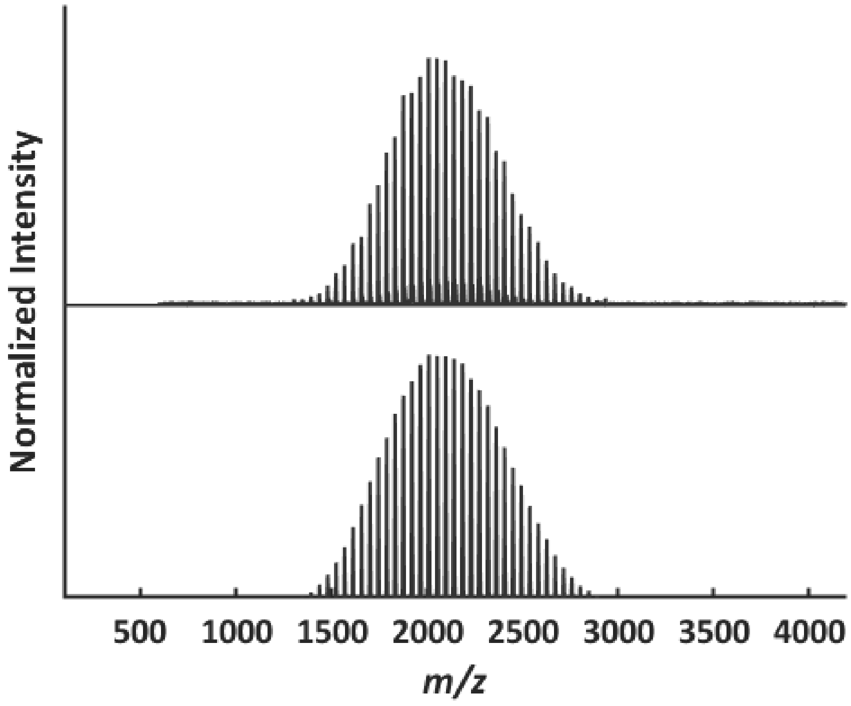

Figure S23. Stacked MALDI-TOF mass spectra of rPEG$^{0.54}_{31}$: (i) crude sample and (ii) sample after purification via ASP. Additional species observed in the crude sample can be attributed to different ionic counterions, without indicating any change in the polymer composition.

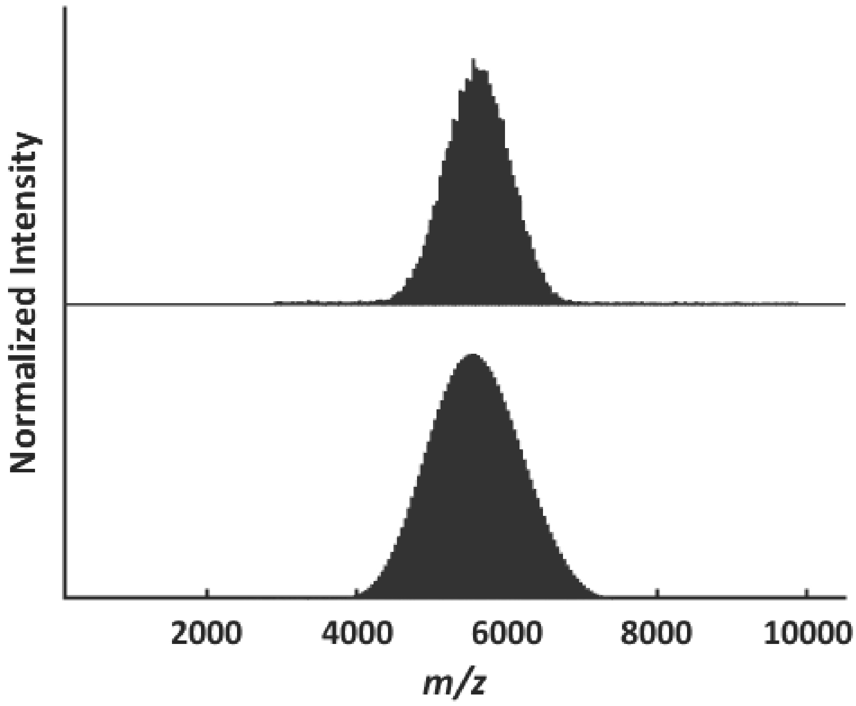

Figure S24. Stacked MALDI-TOF mass spectra of $rPEG_{84}^{0.50}$: (i) crude sample and (ii) sample after purification via ASP.

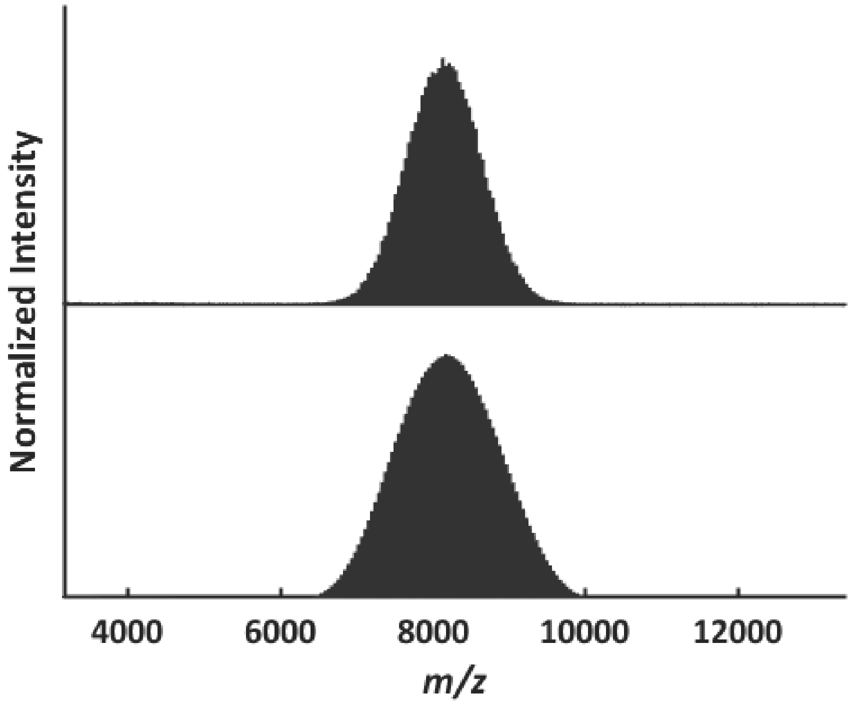

Figure S25. Stacked MALDI-TOF mass spectra of $rPEG_{123}^{0.51}$: (i) crude sample and (ii) sample after purification via ASP.

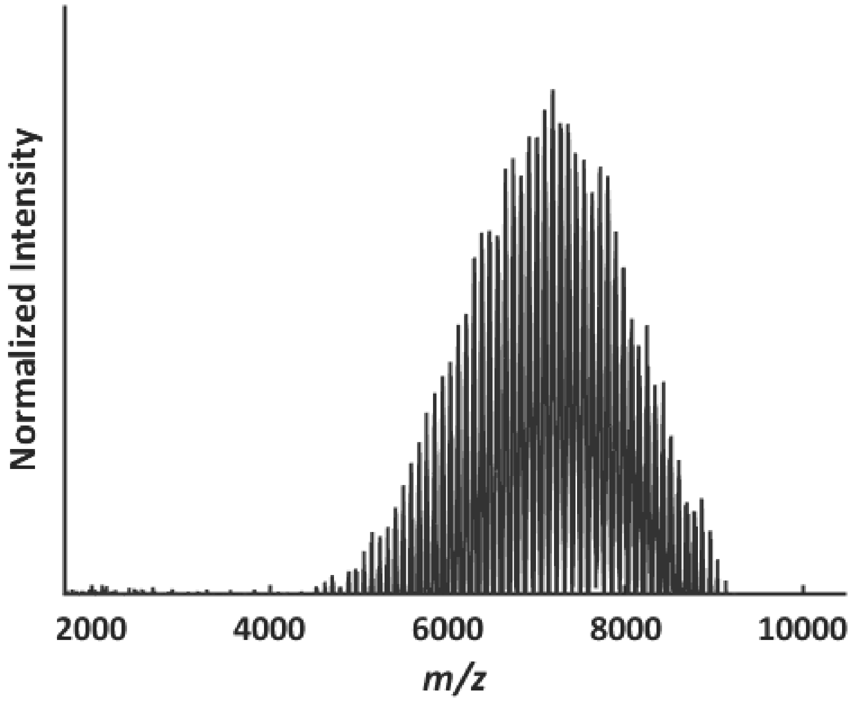

Figure S26. MALDI-TOF mass spectrum of rPEG$^{0.12}_{275}$. Analysis of the crude sample was not possible due to residual impurities.

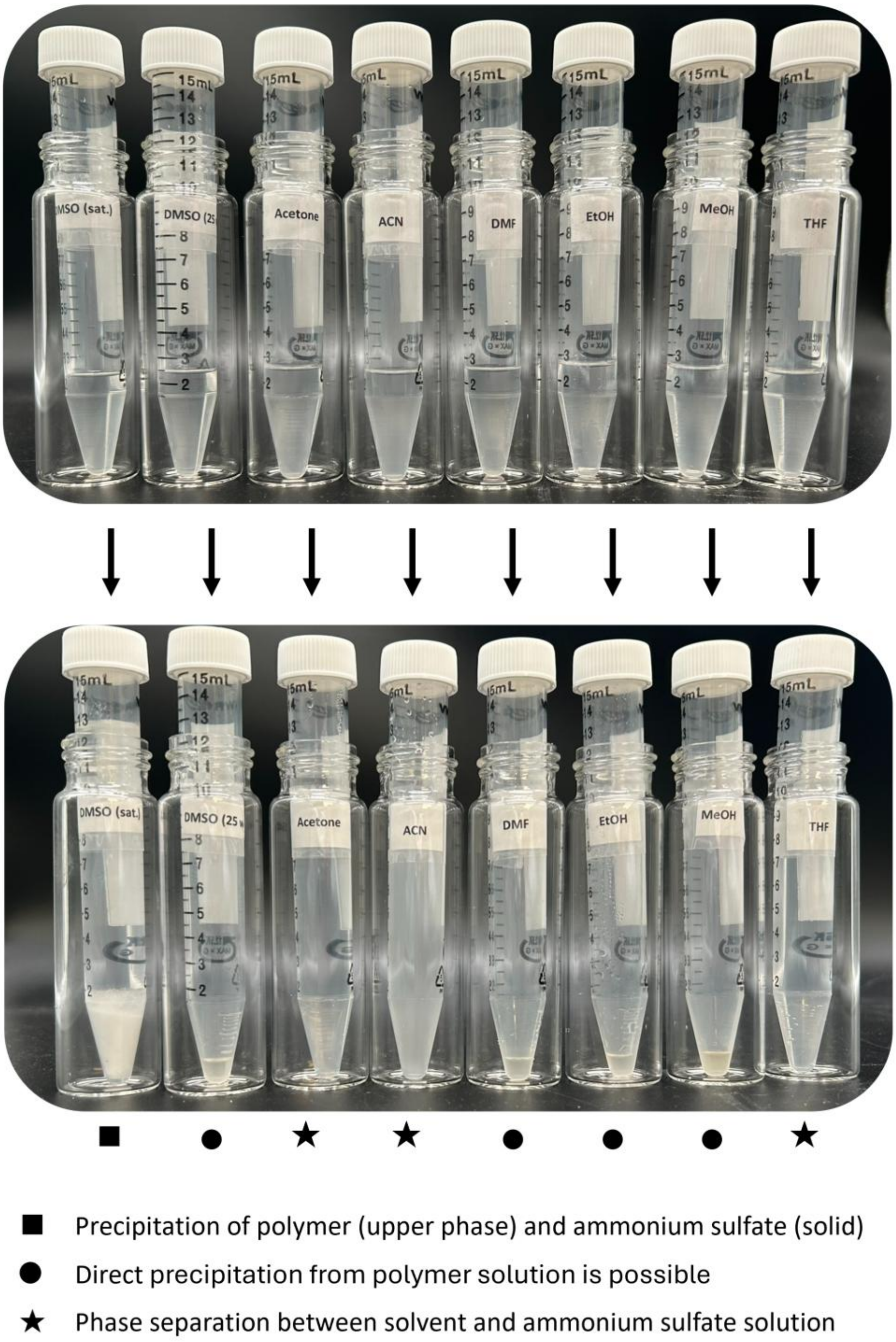

Figure S27. A systematic study was conducted to investigate ammonium sulfate precipitation (ASP) workup of rPEG$^{0.51}_{123}$ from various water-miscible organic solvents, with the aim of evaluating the effect of the solvent on precipitation behavior. The polymer, previously purified, was dissolved in each respective solvent at a concentration of 100 mg mL^{-1} and then subjected to ASP. For one DMSO sample (left), a saturated ammonium sulfate solution (43 wt%) was used for precipitation, resulting in the precipitation of both the polymer and ammonium sulfate (■). For all other samples, a 25 wt% ammonium sulfate solution was used. ASP workup from organic polymer solution was possible for DMSO, dimethylformamide (DMF), ethanol (EtOH) and methanol (MeOH), with the recovered polymer shown in the figure (●). In contrast, for acetone, acetonitrile (ACN) and tetrahydrofuran (THF) solution, addition of ammonium sulfate solution resulted in phase separation between the aqueous and organic phase (★).

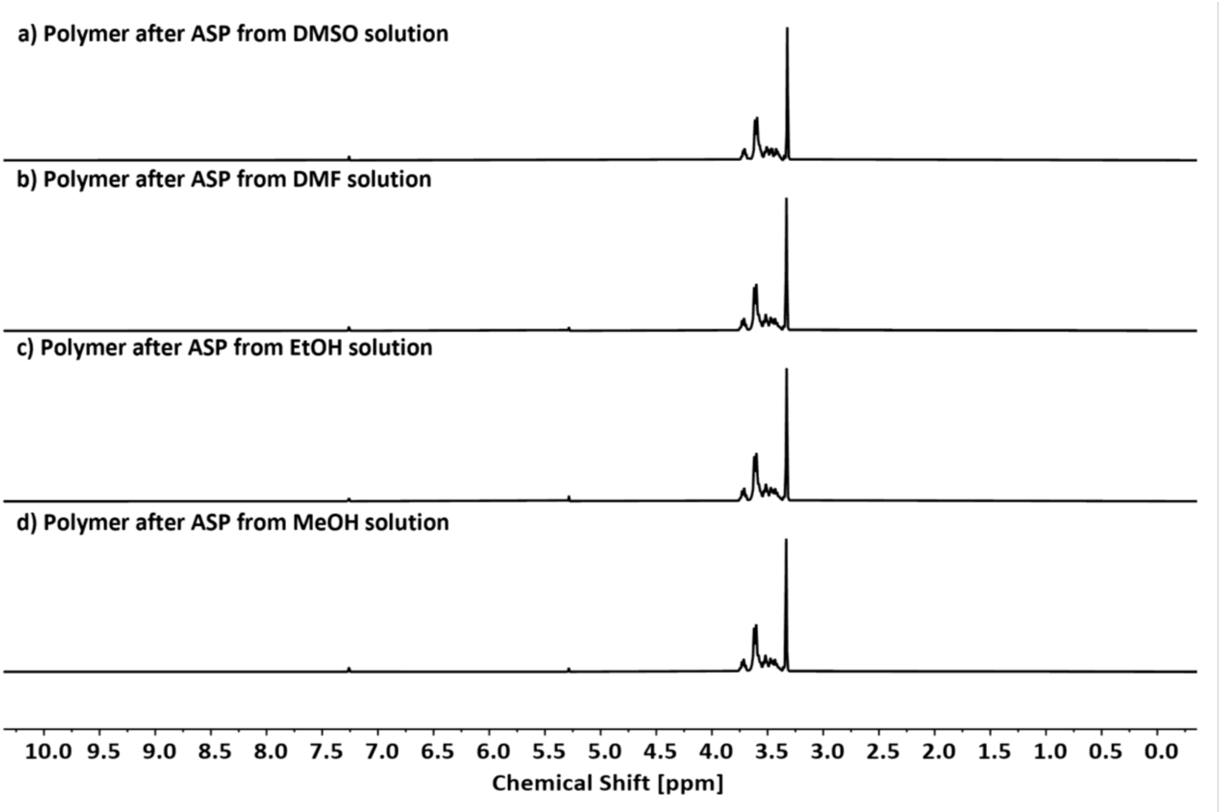

Figure S28. ^{1}H NMR spectra of exemplary polymer ($rPEG_{123}^{0.51}$) purified from different organic solutions (300 MHz, $CDCl_3$): a) ASP from DMSO solution; b) ASP from DMF solution; c) ASP from EtOH solution; d) ASP from MeOH solution.
